# Knotting matters: orderly molecular entanglements

**DOI:** 10.1039/d2cs00323f

**Published:** 2022-08-18

**Authors:** Zoe Ashbridge, Stephen D. P. Fielden, David A. Leigh, Lucian Pirvu, Fredrik Schaufelberger, Liang Zhang

**Affiliations:** Department of Chemistry, The University of Manchester Manchester UK; School of Chemistry and Molecular Engineering, East China Normal University 3663 N Zhongshan Road Shanghai China

## Abstract

Entangling strands in a well-ordered manner can produce useful effects, from shoelaces and fishing nets to brown paper packages tied up with strings. At the nanoscale, non-crystalline polymer chains of sufficient length and flexibility randomly form tangled mixtures containing open knots of different sizes, shapes and complexity. However, discrete molecular knots of precise topology can also be obtained by controlling the number, sequence and stereochemistry of strand crossings: orderly molecular entanglements. During the last decade, substantial progress in the nascent field of molecular nanotopology has been made, with general synthetic strategies and new knotting motifs introduced, along with insights into the properties and functions of ordered tangle sequences. Conformational restrictions imparted by knotting can induce allostery, strong and selective anion binding, catalytic activity, lead to effective chiral expression across length scales, binding modes in conformations efficacious for drug delivery, and facilitate mechanical function at the molecular level. As complex molecular topologies become increasingly synthetically accessible they have the potential to play a significant role in molecular and materials design strategies. We highlight particular examples of molecular knots to illustrate why these are a few of our favourite things.

## Abstract 2

We review recent progress in molecular knotting, the chemistry of orderly molecular entanglements. As complex nanotopologies become increasingly accessible they may play significant roles in molecular design.

## Graphical abstract

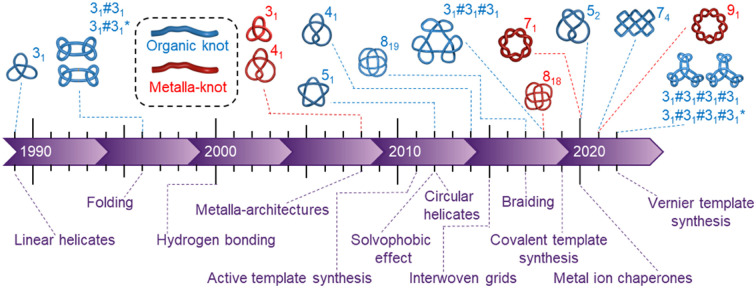

## 1. Introduction

The ability to tie knots marked a major advance in prehistoric technologies, enabling early humans to make tools and materials with new or superior properties (for example, tying axe-heads onto shafts, making fishing nets and weaving fabrics). Even today knots provide solutions to technical challenges, from modern surgical sutures^1^ to restraining cables on the NASA Mars Curiosity Rover.^2^ Knotting and entanglements are also ubiquitous at smaller length scales, including in the structures of DNA,^3^ RNA,^4^ proteins,^5^ synthetic polymers,^6^ liquid crystals^7^ and subatomic particles.^8^ However, the tying of specific knots in molecular strands remains a formidable challenge.^9^ A conceptual synthetic strategy to a molecular knot was outlined as early as 1961,^10^ but it took nearly three decades before the first molecular trefoil (3_1_ in Alexander–Briggs notation^11^) knot was realised by the Sauvage group in 1989.^12^ No other prime knot topologies succumbed to chemical synthesis for a further two decades, the difficulties involved summed up by Sanders and co-workers in a 2012 Science paper: ‘*The synthesis of molecular knots is particularly difficult because it requires precisely defined pathways and transition states that are entropically much more demanding than topologically simpler macrocyclization or catenation processes*’.^13^

**Fig. 1:**
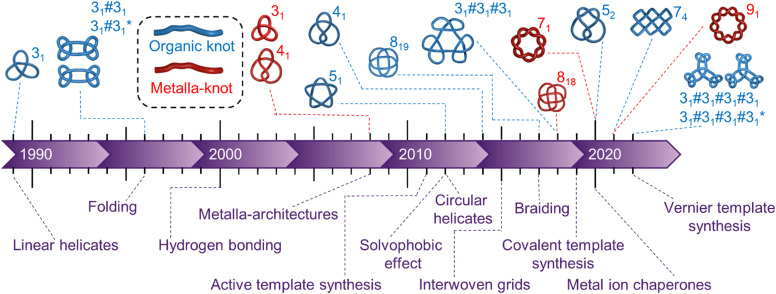
Timeline of the earliest reports of new molecular topologies (above) and major innovations in synthetic strategies to knots (below). ‘Organic knots’ are molecular knots with a continuous backbone of covalent bonds; ‘metalla-knots’††Knots incorporating metal–ligand coordination bonds as an integral part of the strand have been referred to variously in the literature as metallaknots, metallaknotanes, metalla–organic knots, metalloorganic knots and organometallic knots. have metal–ligand bonds as an integral part of the knotted loop. Metal–ligand bonding is often labile, providing a facile mechanism for the strand of a metalla-knot to pass through itself, in conflict with a defining principle of topology in mathematics.

Whilst these issues remain significant, in the last decade strategies have been developed that increasingly allow them to be overcome. Molecular knots have been synthesised with evermore complex topologies (Fig. 1). Metalla-knots, molecules that contain metal atoms as part of the continuous strand backbone, have also become accessible (Fig. 1). Knot synthesis has been aided by the use of interwoven grids,^14–16^ hydrophobic assembly,^13,17–19^ lanthanide ion template synthesis,^20^ and the folding and entanglement of single strands by metal ions in a manner reminiscent of biological chaperones.^21^ Long dreamt of^22^ extended arrays of well-defined tangles have finally become realistic targets for chemical synthesis through molecular weaving^23–26^ and Vernier template synthesis.^27^

With such advances molecular knotting is ready to answer questions regarding nanoscale topology relevant to chemistry, biology and physics. Entangling molecular strands with robust backbones has two important consequences: (i) strand crossing regions cannot pass through each other, blocking pathways to particular conformations and altering molecular dynamics; (ii) the structure becomes non-trivial in topological terms (each crossing can be over or under with respect to others), imparting additional stereochemical complexity. Other mechanically restricted molecules, such as catenanes and rotaxanes can undergo well-defined rearrangements of their components in response to stimuli^28^ or fuelling. The dynamic properties and features of molecular entanglements may ultimately prove similarly significant.

This review outlines the state-of-the-art in the synthesis and what is known of the properties of orderly molecular entanglements.^9*e*^ We focus on synthetic strategies and insights that have recently been disclosed regarding the functions and characteristics of knotted molecular structures. A limited number of catenanes (links) are also included in the review, shown only to highlight similarities and/or differences to molecular knots synthesised by related methods, or when the links occur as side products of knot synthesis.

## 2. Synthesis of molecular knots

### 2.1. Early examples of synthetic molecular knots

**Fig. 2:**
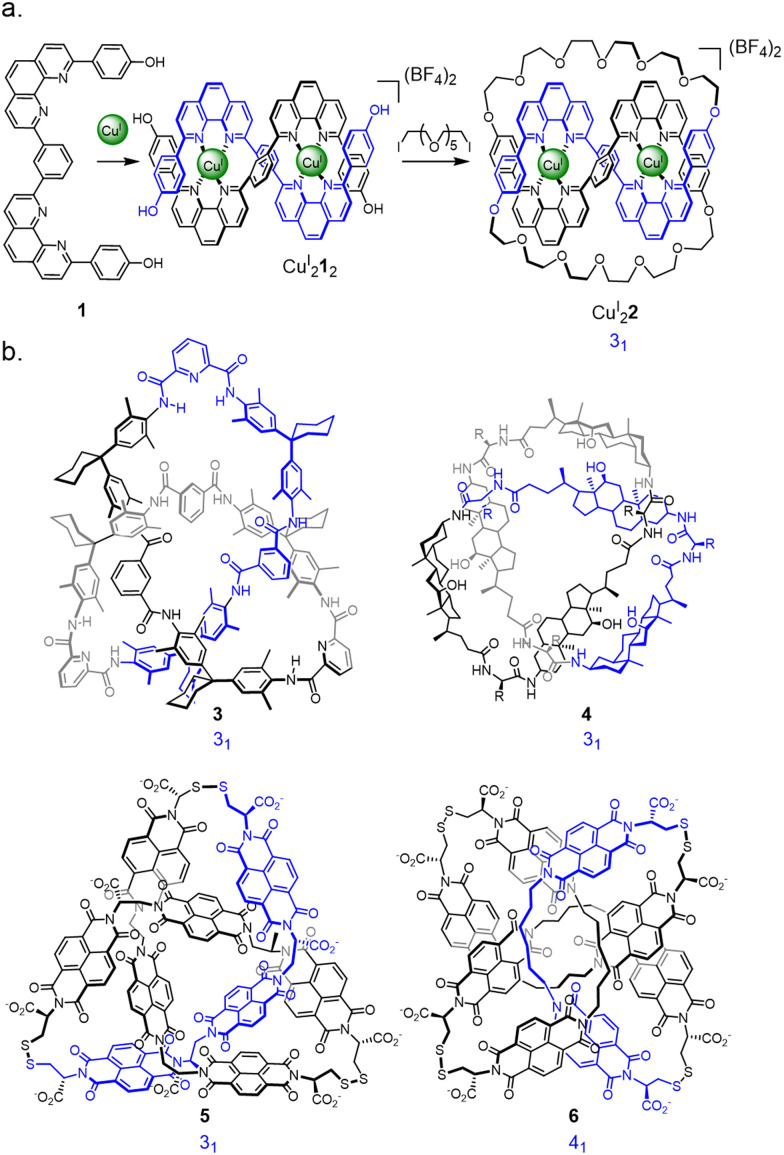
Early synthetic molecular knots. (a) Coordination of Cu^I^ to ditopic ligand 1 to construct trefoil knot Cu^I^_2_2 from a linear helicate.^12^ (b) Examples of molecular knots discovered through serendipity: Hydrogen bond assembled knot 3, first prepared by Hunter *et al.*^31^ but structurally reassigned as a knot following X-ray crystallography by Vögtle *et al.*^30^ Hydrogen bond assembled steroidal knot 4 from Feigel *et al.*^32^ Disulfide-linked trefoil knot^13^5 and figure-eight knot^17^6 discovered by Sanders *et al*.

Extrapolating from the seminal metal template synthesis of catenanes,^29^ the Sauvage group entwined two ligand strands (1) in a linear helicate Cu^I^_2_1_2_ such that the strands cross each other three times with over-under-over relative stereochemistry (Fig. 2a).^12^ Macrocyclisation of the four termini along the length of the helicate afforded the trefoil-knotted complex Cu^I^_2_2, together with a number of other products (*e.g.* the topologically-trivial unknot macrocycle) resulting from different regiochemical strand connections.^12*a*^

The use of metal templates to organise ligand strand crossings, followed by covalent capture of the entangled structure, is an attractive principle for several reasons: (i) coordination to metal templates is often dynamic, which enables ‘error checking’ during the assembly to generate the required entanglements under thermodynamic or pseudo-thermodynamic control.^9^ (ii) The template can often be removed after covalent capture of the closed loop, which prevents the resulting wholly organic knot from untying. (iii) Metal helicates have an additional useful property, as every metal centre within the same helicate has the same stereochemistry, imparting a defined crossing sequence if the strands ends are connected in the right way.

A number of the early knot-forming reactions were unanticipated discoveries (Fig. 2b). From an X-ray crystal structure Vögtle was able to reassign^30^3 as a trefoil knot, a compound that had originally been reported^31^ to be a hydrogen bond assembled [2]catenane. Feigel discovered another hydrogen bond assembled trefoil knot 4, derived from steroidal peptide building blocks.^32^ Dynamic disulfide exchange of hydrophobic components was found by Sanders to unexpectedly form a trefoil knot^13^5 and figure-eight (4_1_) knot^17^6. (More recently, the hydrophobic effect has been used by Cougnon as a deliberate, if as yet somewhat unpredictable, strategy to access knotted and linked structures, see Section 2.9.^18,19^) These early largely unexpected examples of molecular knots gave some indications into how the synthesis of topologically complex structures might be tackled. However, the evolution of more rational syntheses of molecular knots has been greatly aided by advances in synthetic methodology, instrumentation, characterisation methods, and the consideration of tangle theory in molecular design.

### 2.2. Designing and connecting tangles

The process of knotting a strand has different requirements at different length scales. When tying a shoelace, we rely on friction and inertia to hold the strand in the entangled conformation. But such effects are not significant at the nanoscale and so the construction of molecular knots must rely on inter/intramolecular forces to direct entanglement. It follows that most synthetic molecular knots are either formed as a thermodynamically stable product or are derived from the covalent capture of a similarly stabilised complex.

Symmetry is a common feature of many of the orderly molecular entanglements prepared to date. Most knots have been synthesised based on direct consideration of the requisite symmetry (threefold for a trefoil knot, fivefold for a pentafoil knot, *etc.*) or by targeting particular symmetric representations of a knot (as for the figure-eight knot 6 in Fig. 2b). This has led to several theoretical treatise on the generation of symmetric knots, such as torus knots.^22*a*,33^ To design syntheses of less symmetric entanglement patterns in a rational manner it is helpful to consider aspects of knot theory.^34^

Mathematically a knot is a closed loop with a topology that cannot be changed by continuous deformation. A molecular knot is hence a self-entangled macrocycle where non-nugatory^9*e*^ crossings cannot be removed without cleaving the covalent backbone. The mechanical restrictions imparted on the strand makes molecular knots close relatives of mechanically interlocked molecules, such as catenanes (‘links’ in mathematics) and rotaxanes (which are topologically trivial but the intrinsic restrictions in bond lengths and geometries keep the components mechanically associated nonetheless).^28^ Mathematicians treat knots as 1D strands with no restrictions on how the strand can be twisted or turned, but for molecules it is important to bear in mind both mathematics and the physical laws of chemistry (*i.e.* strain, bond length, *etc.*) when considering the properties and features of entanglements.^35^

**Fig. 3:**
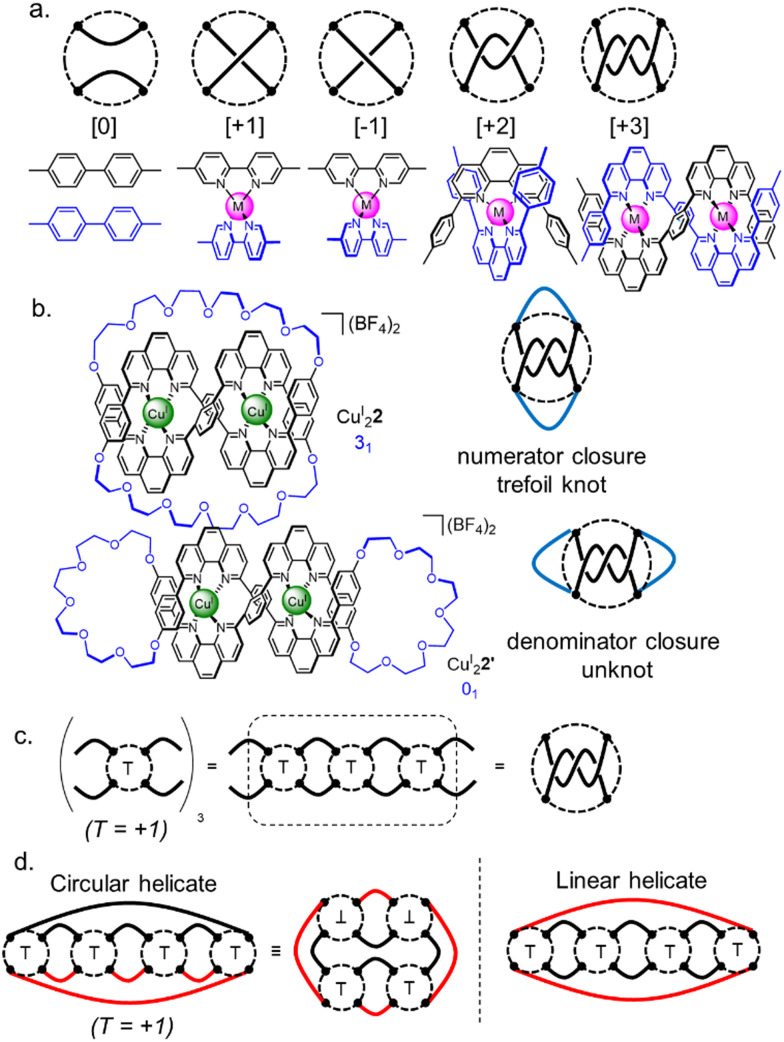
Tangle theory applied to molecular nanotopology.^36^ (a) Examples of simple tangles and their chemical equivalents. (b) Possible tangle closures. (c) Summation of tangles to form larger tangle sequences. (d) Circular helicate and linear helicate strategy to a Solomon link illustrated with tangles. Black full lines indicate pre-formed closures, red lines indicate closures required after self-assembly of an intermediate containing most of the required tangles and building block connections.

There are an infinite number of possible knots and links, so a theory that describes their structural relationship in terms of smaller fragments is helpful. Such a system, tangle theory, was introduced by Conway in the 1970s.^36^ A tangle is a portion of a knot containing two 1D parts of the strand (‘strings’) within an imaginary circle that the strands cross four times. Connections between strings occur outside circles, whilst crossings occur inside. Some simple tangles and their chemical equivalents are shown in Fig. 3a. Combining tangles and closing the resulting strings gives a knot or link. Horizontal connection of the strings is termed a numerator closure, whilst vertical connection gives a denominator closure (Fig. 3b). The fragments that form tangles are the equivalent of chemical synthons, so tangle theory can help in the design of a synthesis of a particular topology.

Early work on metal template knot synthesis focused on parameters such as coordination number (*e.g.* 4, 6, 9), coordination geometry (*e.g.* octahedral, tetrahedral, linear) and angle of divergence (‘turn angle’) within crossing points.^22*a*,37^ This provided a collection of motifs that can be used as synthons for complex topologies. As template strategies for orderly entanglements have evolved, the focus has shifted from tuning individual crossing points towards controlling their connectivity. Polytopic ligands containing different binding sites and multiple functionalities, and thus sequence information, have been used to generate increasingly complex topologies. Joining tangles (*i.e.* crossing points) gives a rationally designed tangle sequence (Fig. 3c and d) which may then be closed to give a particular given knot.

The use of tangles as synthons in molecular nanotopology proceeds according to the following algorithm:

(1) Map crossings in terms of tangles.

(2) Establish absolute stereochemistry of each tangle.

(3) Establish relative stereochemistry in the tangle sequence.

(4) Define sequence information, *i.e.* ligand connectivity.

(5) Define tangle closures, *i.e.* geometrical information regarding strand proximity.

**Fig. 4:**
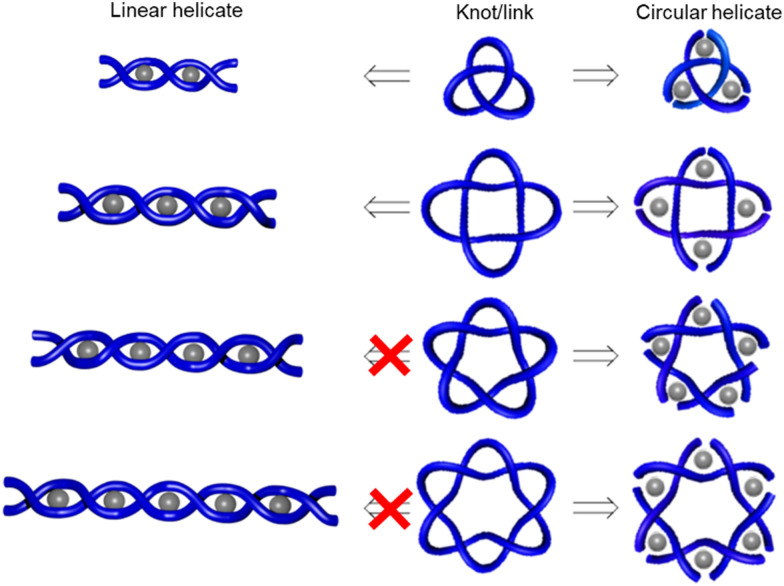
Linear and circular helicate strategies to knots and links.^9*e*^

To illustrate this we can consider the metal-template trefoil knot synthesis of the Sauvage group in terms of tangle theory:^12^ the racemic linear helicate generated by complex formation between two Cu^I^ ions and two molecules of bidentate ligand 1 maps out the [±3] tangle (*i.e.* a racemic mixture of helicates). A numerator closure (Fig. 3b) yields trefoil knot Cu^I^_2_2 whilst a denominator closure gives an unwanted byproduct, the topoisomeric unknot macrocycle Cu^I^_2_2′. This exemplifies the need to design systems that generate both the required sequence of crossings (*i.e.* the correct tangles) and a conformation that favours the necessary numerator or denominator closures. With extended ligands, the linear helicate strategy proved amenable for the synthesis of a Solomon 4^2^_1_ link from a complex mapping the [±4] tangle (Fig. 4).^38^ However, as the length of the helicate increases making numerator *versus* denominator connections becomes ever more difficult to control, making linear helicates less useful for more complex knots or links.^39^

To overcome the limitations of the linear helicate strategy, circular helicates have been used to generate higher-order knots and links.^9*e*,40^ The self-assembly of oligodentate bipyridine (bipy) ligands and metal ions into pentameric and hexameric circular helicates was serendipitously discovered by the Lehn group in the 1980s.^40^ Circular helicates, and related ligand systems, are effective scaffolds for the generation of higher-order knots and links, as each overlap of the ligands produces a crossing point (a [±1] tangle). The well-defined structures of these complexes can be organised such that particular regiochemical closures are favoured over others, by designing strands whose conformations either bring the end groups into close proximity for the required connection or simply preclude other connections. This corresponds mathematically to the stepwise connection of a 
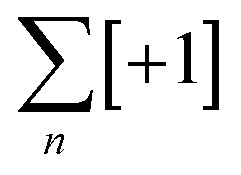
 or 
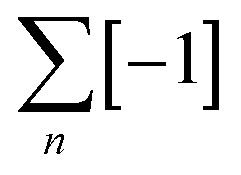
 tangle sequence, rather than the numerator closure of the corresponding [*n*] tangle (Fig. 3d). The drawback of this approach—the increased number of closures required to yield the fully interlocked architecture—is overcome by using dynamic ‘error checking’ chemistries for the ligand connections. The closed knot (or link) topology is therefore generally the thermodynamically favoured product.

### 2.3. Knots and links derived from circular metal helicates and molecular cages

#### 2.3.1. Covalent capture of circular helicates by imine formation

**Fig. 5:**
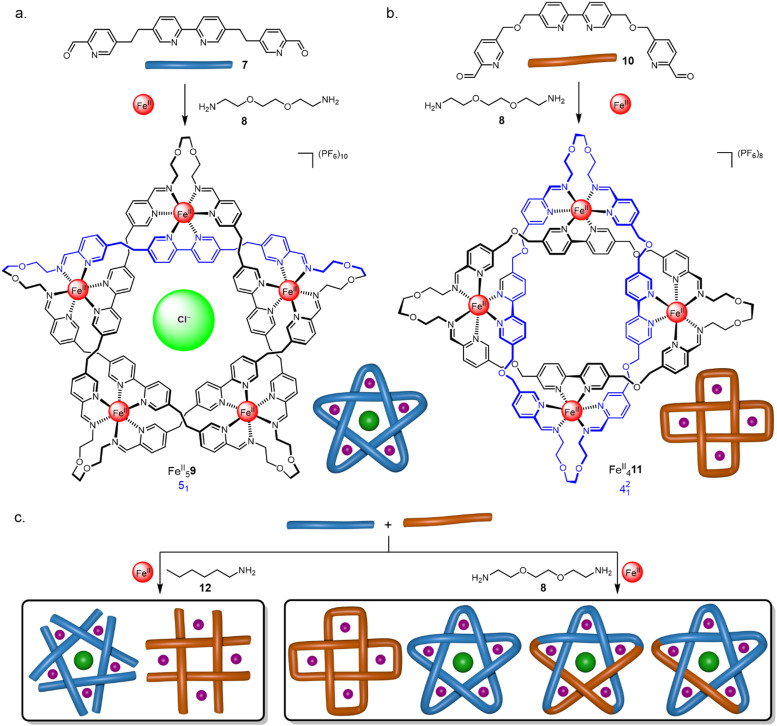
Self-assembly of (a) molecular pentafoil knot^43^ Fe^II^_5_9 and (b) Solomon link^45^ Fe^II^_4_11 using dynamic imine exchange. (c) Self-sorting of circular helicates, knots and links.^46^

An error-checking reaction frequently used for closing circular helicates and related metal-coordination scaffolds is imine bond formation between aldehydes and amines. In terms of mechanically interlocked molecule synthesis, imine formation was originally combined with metal coordination to access catenane Hopf (2^2^_1_) links^41^ under thermodynamic control and subsequently applied to molecular Borromean rings^42^ (6^3^_2_ link). The first molecular pentafoil (5_1_) knot was prepared by this strategy in 2012 (Fig. 5a).^43^

The generation of a pentafoil knot requires five sets of building block connections from a pentameric circular helicate (Fig. 5a). Bis-aldehyde 7 is related to Lehn-type tri(bipy) ligand strands with the outer pyridine of each terminal bipy group replaced by an aldehyde which is converted to a coordinating imine by the reversible condensation reaction with an amine. Open imine circular helicates readily assembled when 7 was mixed with a range of monomeric aliphatic and benzylic amines and FeCl_2_ in DMSO. X-Ray crystallography showed a chloride ion tightly bound within the cavity of the helicate by 10 C–H⋯Cl^−^ hydrogen bonds, consistent with chloride acting as a template for the circular helicate. The helicate assembly proved sensitive towards reactant stoichiometry, solvent and concentration.^44^ With bis-amine linker 8, the ligands were connected to give the corresponding pentafoil knot Fe^II^_5_9 in 44% yield. The oligo(ethylene glycol) bridge of the bisamine proved crucial for knot formation as it favours a gauche conformation stereoelectronically, facilitating the turn required in the connecting loop. In contrast, alkyl chain bis-amines gave polymeric mixtures and no evidence of knots. The X-ray crystal structure of Fe^II^_5_9 indicates that if the glycol oxygen atoms of the chain were replaced with CH_2_ groups then 1,3-diaxial clashes of the C–H groups would also disfavour cyclization of the loop.

Ligand 10, containing an additional oxygen atom in the two-carbon bridge, forms a tetrameric circular helicate with monoamines.^40^ The corresponding Solomon link Fe^II^_4_11 was obtained in 75% yield with bis-amine 8 (Fig. 5b).^45^

Reflecting the selective formation of tetra- and pentameric helicates from ligands 10 and 7, respectively, combining both dialdehydes with hexylamine 12 and FeCl_2_ led to perfectly self-sorted tetrameric and pentameric circular helicates (Fig. 5c).^46^ Mixing both ligands with diamine 8 produced Solomon link Fe^II^_4_11 and pentafoil knot Fe^II^_5_9, as well as a small amount of the pentafoil knots with one or two ligands of 7 substituted by 10. This indicates that the closed loop complexes constitute a kinetic trap, as mixing of preformed closed species Fe^II^_5_9 and Fe^II^_4_11 under similar conditions did not lead to scrambling.^47^ Dynamic imine chemistry and metal templates have also been used by other groups^48–50^ to direct entanglement formation. For example, Trabolsi has developed metal template routes to imine trefoil knots and Solomon links (see Section 4).^48^

#### 2.3.2. Covalent capture of circular helicates by ring-closing alkene metathesis

Whilst imine condensation is an effective means of connecting building blocks organised on scaffolds through metal coordination, the lability of imine bonds means the strands can (reversibly) reopen upon demetallation. This results in the demetallated knot unravelling unless it is thermodynamically stable without metal ion coordination. It can be difficult to ‘trap out’ knots from coordinated complexes by reducing imine groups to kinetically robust amines. This is likely because each imine reduction successively weakens the metal complex and so it disassembles, and the strand unravels, before the entire knot can be covalently captured through amine linkages.

Other strand-connecting reactions featuring less dynamic chemical bonds have been used to join building blocks organised on metal-coordinated scaffolds. Olefin metathesis has proved amongst the most useful in this respect, as the alkene connections are only dynamic (useful for error correction of building block connections) in the presence of a catalyst.^51^

**Fig. 6:**
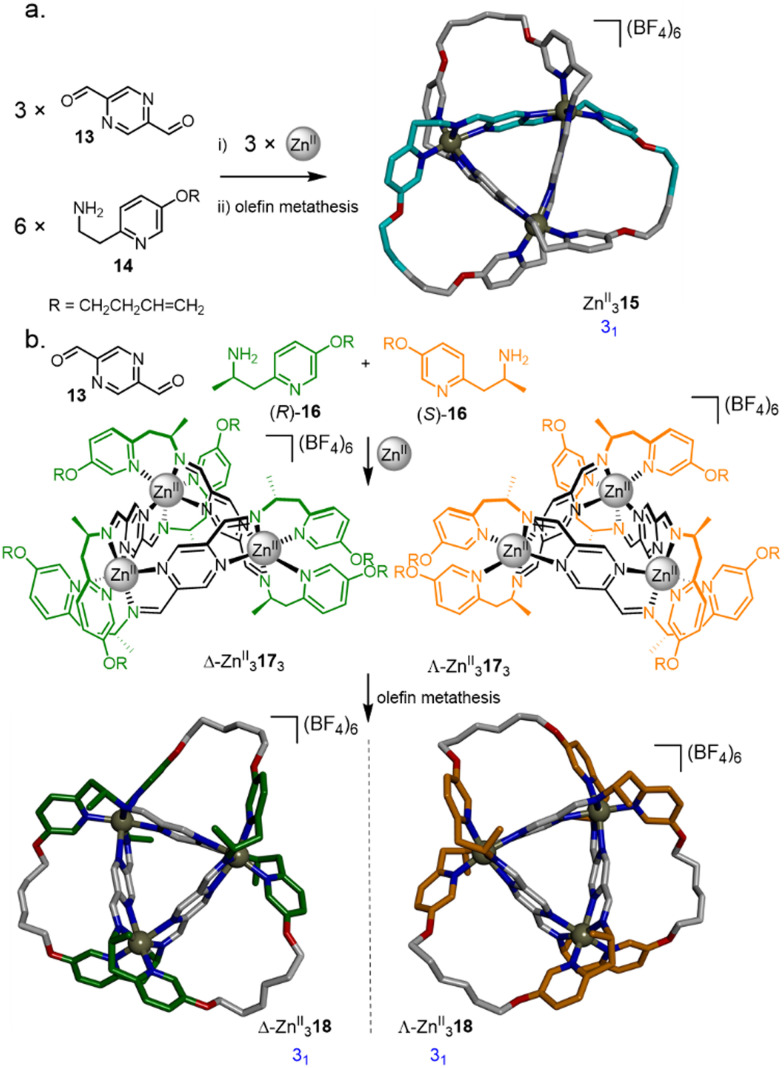
(a) Synthesis of molecular trefoil knot Zn^II^_3_15*via* a trimeric circular helicate.^53^ (b) Chiral self-sorting of trefoil knots based on a pyrazine-2,5-dicarbaldehyde motif.^54^

The synthesis of a trefoil knot *via* a trimeric circular helicate by olefin metathesis was achieved through a modification of a complex reported by Brooker *et al.*,^52^ assembled from Zn^II^(BF_4_)_2_, pyrazine-2,5-dicarbaldehyde 13 and pyridine 14 (Fig. 6a).^53^ Closure of the circular helicate to form racemic knot Zn^II^_3_15 by olefin metathesis proceeded in 90% overall yield. Use of enantiopure amine (*R*)-16 or (*S*)-16 directs the stereoselective formation of either Λ-Zn^II^_3_17_3_ or Δ-Zn^II^_3_17_3_ giving, after olefin metathesis, the respective knots Λ-Zn^II^_3_18 and Δ-Zn^II^_3_18 (Fig. 6b).^54^ Using a 1 : 1 mixture of (*R*)-16 and (*S*)-16 gave a self-sorted mixture of Λ-Zn^II^_3_17_3_ and Δ-Zn^II^_3_17_3_, with no diastereomeric scrambling observed.

**Fig. 7:**
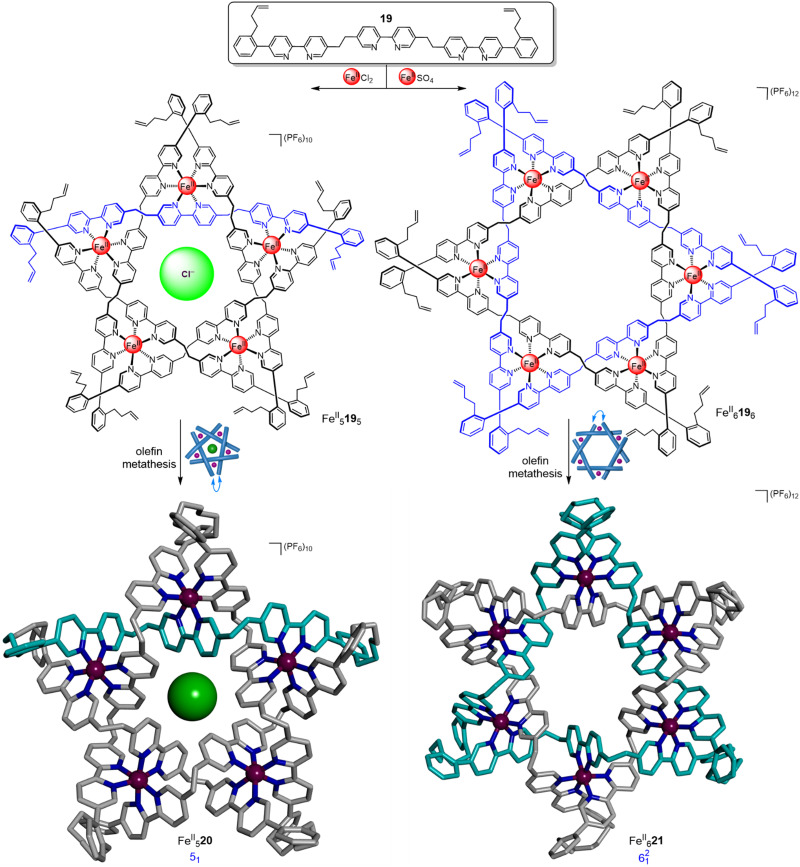
Generation of hexameric and pentameric circular helicates and their subsequent closure by olefin metathesis to form a pentafoil knot Fe^II^_5_20^55^ and a Star of David triply interlocked [2]catenane Fe^II^_6_21.^56^

Using a similar approach but from pentameric and hexameric circular helicates, respectively, a molecular pentafoil knot and a molecular Star of David link (6^2^_1_, a [2]catenane composed of two triply-entwined macrocycles) were obtained (Fig. 7).^55,56^ Ligand 19 generated pentameric circular helicate Fe^II^_5_19_5_ in 89% yield when FeCl_2_ was used.^55^ The corresponding molecular pentafoil knot, Fe^II^_5_20, was obtained in 98% yield through alkene metathesis using the Hoveyda–Grubbs 2nd generation catalyst. A key feature of the building block design is the conformational restriction of the alkene-terminated chains of 19 achieved by attaching them to the *ortho*-position of a phenyl ring, reducing the conformational space accessible to the ligand termini. Without this feature, for example using oligo(ethylene glycol) or alkyl chains, no knot was obtained. Unlike imine-metal-coordinated knots and links, demetallation of Fe^II^_5_20 proceeded smoothly to generate 20, a wholly organic molecular pentafoil knot.

In the absence of a chloride template, hexameric circular helicates are the thermodynamic product from reaction of these types of ligand strands with metal salts.^40^ Hexameric circular helicate Fe^II^_6_19_6_ was formed in 72% yield from the self-assembly of ligand 19 and FeSO_4_ (Fig. 7).^56^ Connection of the ligand termini by olefin metathesis gave Star of David catenane Fe^II^_6_21 in 92% yield. This complex could also be smoothly demetallated to give the fully organic molecular link 21.

The robustness of metal-free pentafoil knot 20 allowed its coordination chemistry to be investigated.^57^ Distinct complexes of knot 20 containing Fe^II^, Co^II^, Ni^II^ or Cu^II^ metal ions could not be obtained *via* self-assembly of 19 or direct remetallation of 20. However, they could all be accessed by transmetallation of Zn^II^_5_20 with the corresponding M^II^(BF_4_)_2_ salts, suggesting that labile Zn^II^ ions pre-organize the relative positions of the bipyridyl binding sites. Stepwise exchange of the Zn^II^ ions for other metal ions can then proceed without generating ‘mistakes’ in the coordination mode of the knotted strand, which would be slow to correct with less labile metal ions. The affinities of the metallated knots towards chloride anions was measured with isothermal titration calorimetry, with Fe^II^_5_20, Co^II^_5_20 and Ni^II^_5_20 having similar binding affinities (*K* ∼ 10^7^ M^−1^) whilst Zn^II^_5_20 (*K* ∼ 10^6^ M^−1^) and Cu^II^_5_20 (*K* ∼ 10^4^ M^−1^) exhibit lower affinities for chloride ion. The differences likely reflect the effect of the different metal ion coordination geometries on the knot conformation, and therefore the size, shape and electronics of the central chloride binding cavity.

**Fig. 8:**
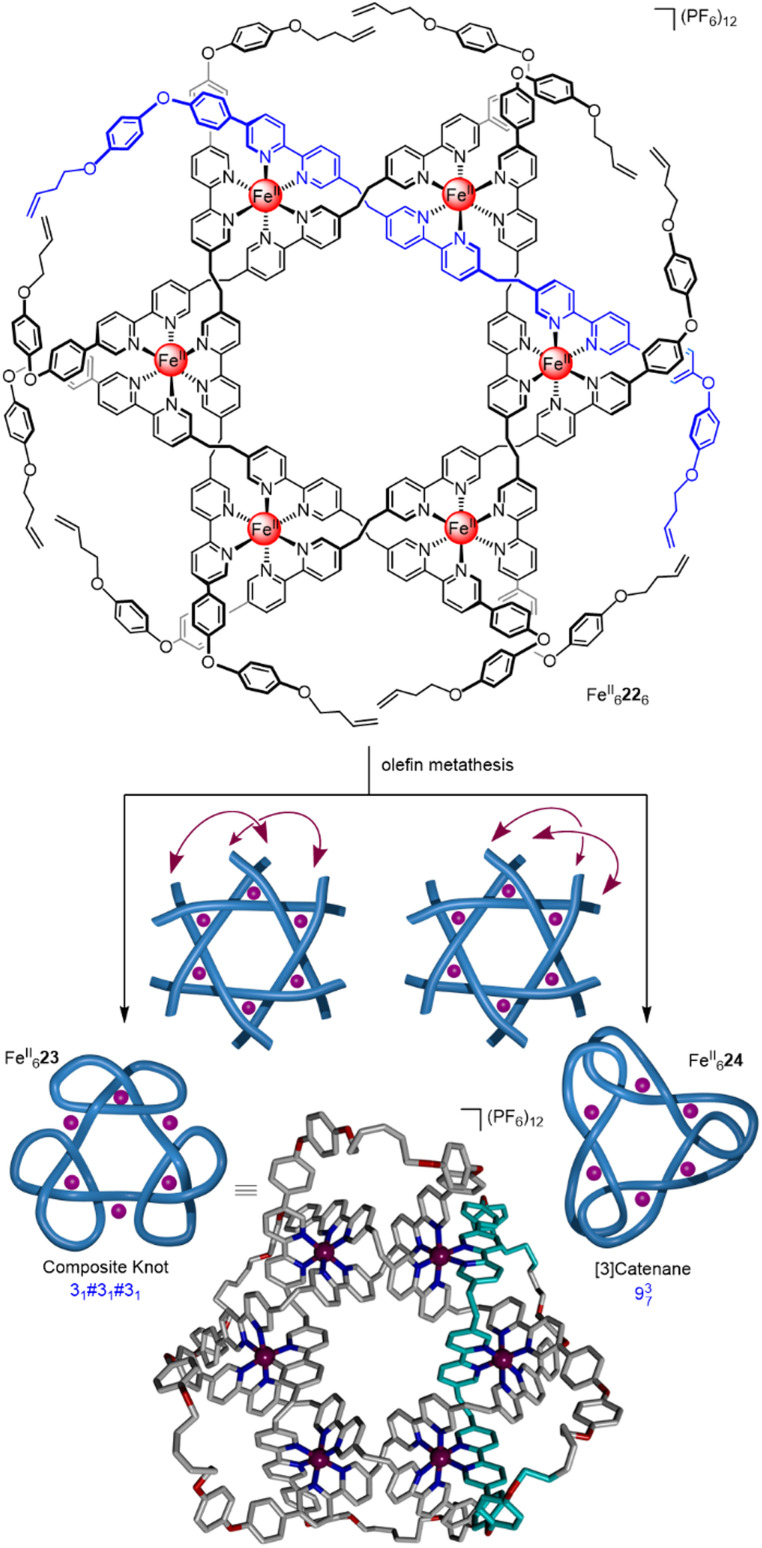
A hexameric circular helicate and its closure to give molecular composite knot Fe^II^_6_23 and link Fe^II^_6_24.^58^

By designing the ligand strands so that they were organised to make different regiochemical connections, a hexameric circular helicate gave access to a composite knot (a knot topologically derived from linear combinations of ring-opened prime knots) containing nine alternating crossings (Fig. 8).^58^ Connecting the ligands of hexameric circular helicate Fe^II^_6_22_6_ by olefin metathesis gave racemic (+3_1_#+3_1_#+3_1_ and −3_1_#−3_1_#−3_1_) composite knot Fe^II^_6_23 (*i.e.* a knot consisting of three trefoil entanglements of the same handedness joined together). However, each ligand terminus in Fe^II^_6_22_6_ is equidistant to two inequivalent ligands. This meant that another topology, 9^3^_7_ link Fe^II^_6_24, was also formed in equal amounts to the knot. Knot Fe^II^_6_23 was separated from the link isomer by crystallisation. Analysis by X-ray crystallography confirmed that the 324-atom loop of Fe^II^_6_23 crosses itself nine times with the same handedness at all six metal centres.

#### 2.3.3. Molecular braiding

**Fig. 9:**
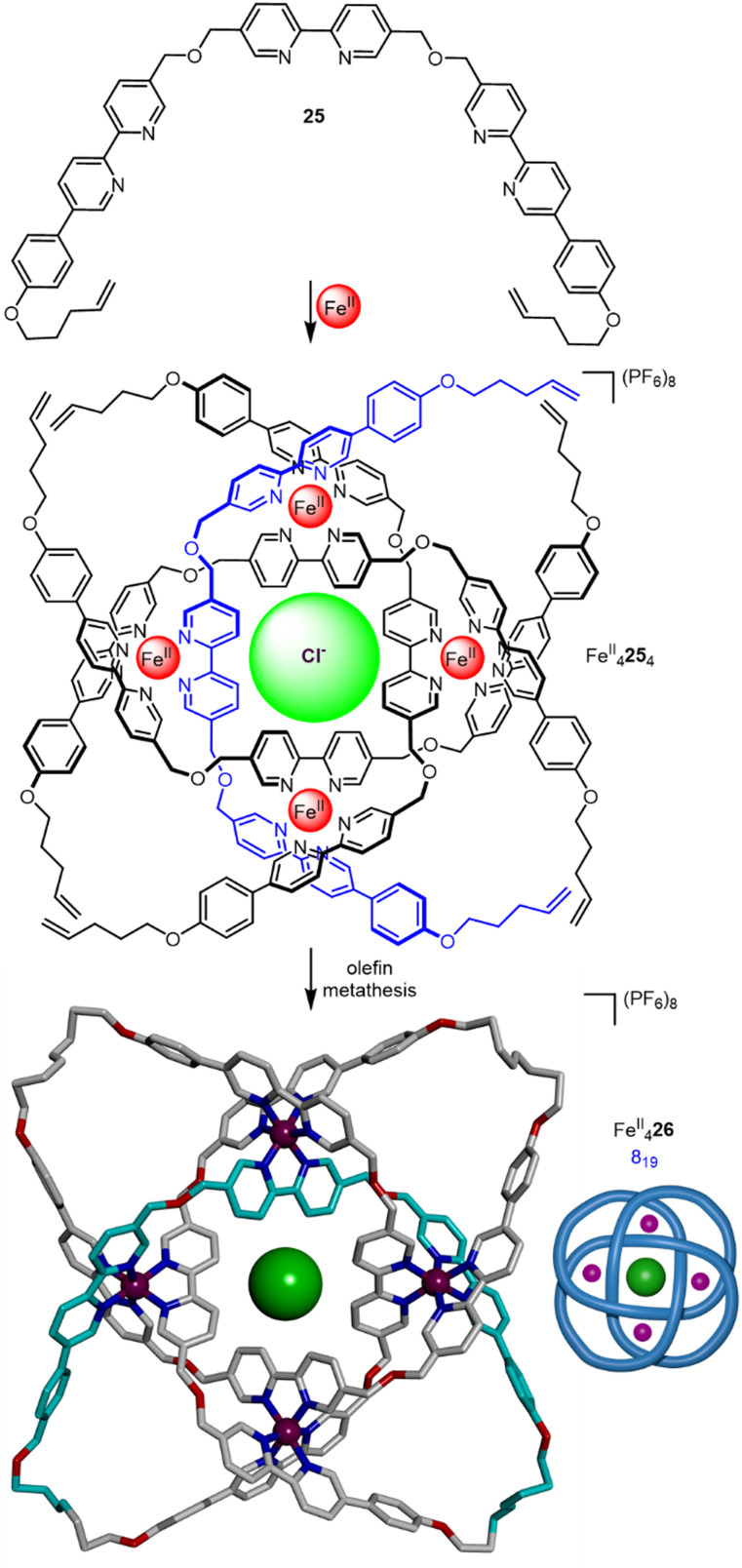
Synthesis of 8_19_ knot Fe^II^_4_26*via* braiding of three ligand strands.^59^

As the octahedral metal ions used in the circular helicate strategy can each bind three bidentate groups, the potential exists for each metal to control the relative positions of—and thereby braid—three strands. Ligand 25 self-assembles with FeCl_2_ in 60% yield to form a circular tetrameric helicate where each metal coordinates to three separate ligand strands (Fig. 9). Closure of the helicate yields 8_19_ knot Fe^II^_4_26, in 62% yield. Unlike the repeating over-under crossing sequence of the 3_1_ and 5_1_ knots, the 8_19_ knot has a repeating over-over-under-under crossing sequence, a molecular non-alternating knot.^59^ The 8_19_ knot was demetallated and the two topological enantiomers were separated by HPLC. The 192-atom long knotted strand crosses itself eight times in the closed loop, making 26 the tightest molecular knot recorded to date.

#### 2.3.4. Knots derived from molecular cages

**Fig. 10:**
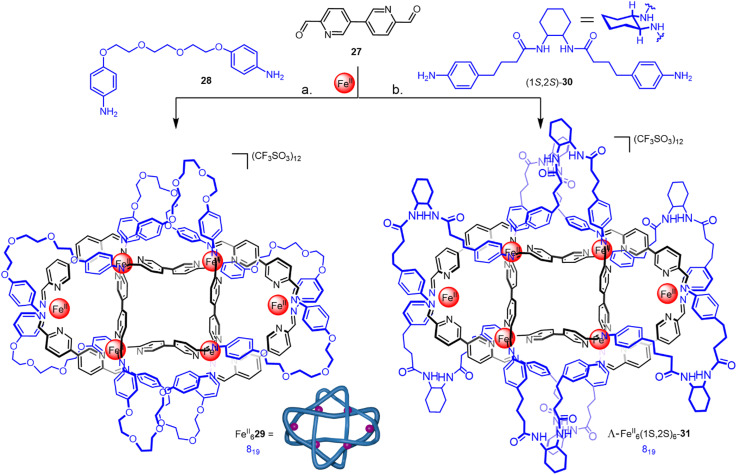
(a) Synthesis of racemic 8_19_ knot Fe^II^_6_29 from dianiline 28. (b) Synthesis of 8_19_ knot Λ-Fe^II^_6_-(1*S*,2*S*)_6_-31 from enantiopure dianiline (1*S*,2*S*)-30.^60^

Molecular cages assembled through metal–ligand coordination often have well-defined three-dimensional shapes. In a similar manner to circular helicates, the intrinsic symmetry of molecular cages can be used to template the formation of knots and links. Nitschke and coworkers have used imine–metal coordination (see Section 2.3.1) to synthesize a range of intricate higher-order catenanes from molecular cage structures.^50^ Most recently, they have utilised dynamic imine formation to synthesize an 8_19_ knot Fe^II^_6_29 from dialdehyde 27 and dianiline 28 (Fig. 10a).^60^ The metallated structure possesses *D*_2_ symmetry, with two distinct environments for the eight glycol linkers and for the six metal centres. Imine reduction and demetallation gave the metal-free 8_19_ knot. Related knot Λ-Fe^II^_6_-(1*S*,2*S*)_6_-31 was prepared diastereoselectively *via* enantiopure dianiline (1*S*,2*S*)-30 (Fig. 10b).

Other early examples of entangled architectures derived from cages include a ‘double trefoil knot’ from the Clever group^61^ and a universal 3-ravel from Lindoy.^62^ These structures are metalla-architectures, with metal–ligand bonds as an intrinsic part of the topology, rather than molecular knots with a continuous backbone of covalent bonds (see Section 2.10). The Fujita group has reported a series of entangled cage architectures, including a double-walled cage capable of guest-adaptive molecular recognition,^63^ and a series of interconvertible entangled cages driven by weak secondary π-acetylene interactions.^64^ These architectures display unusual co-crystallisation behaviour^65^ and show potential for enantioselective catalytic and host–guest applications.^66^

### 2.4. Lanthanide template synthesis of knots

**Fig. 11:**
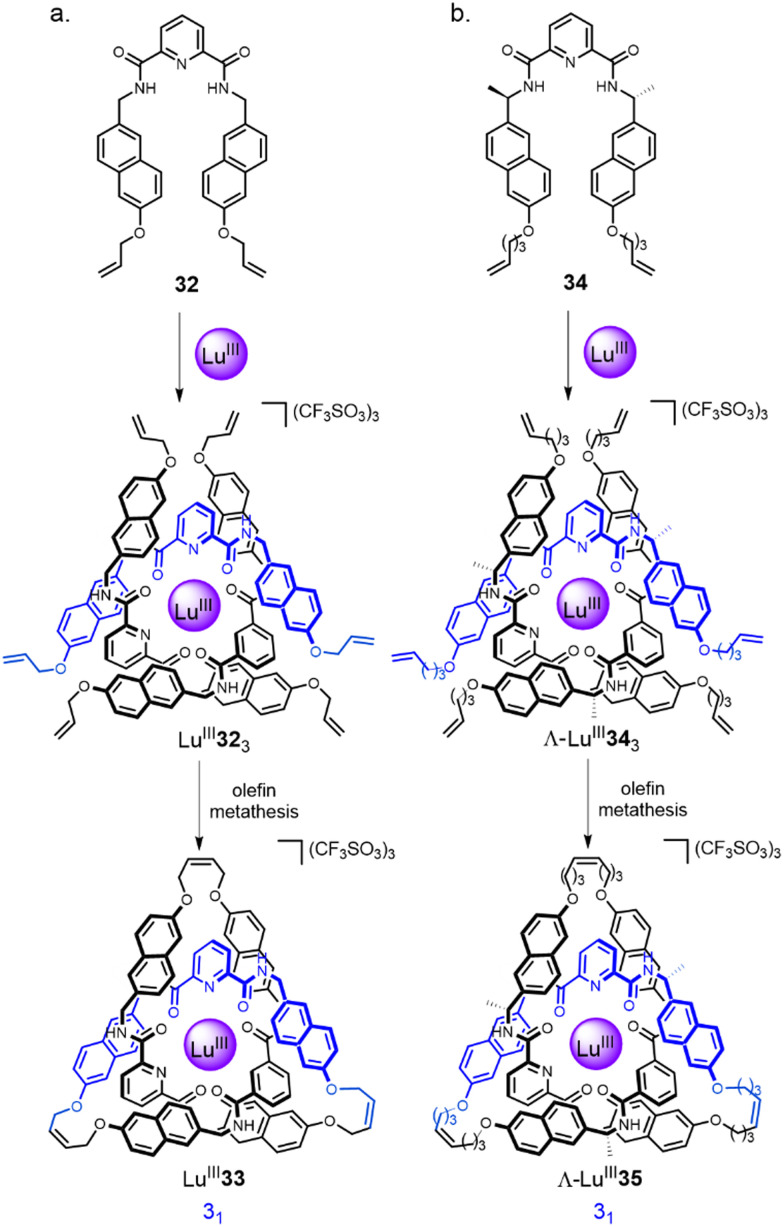
Synthesis of: (a) trefoil knot Lu^III^33*via* racemic circular helicate Lu^III^32_3_;^20^ (b) trefoil knot Λ-Lu^III^35*via* enantiopure circular helicate Λ-Lu^III^34_3_.^70^

A class of Ln^III^ complexes incorporating a 3 : 1 ligand : metal ratio has been extensively investigated by the Gunnlaugsson^67^ and Piguet^68^ groups, and others, over the past two decades. The Leigh group have developed these lanthanide template systems for the formation of knots. The initial approach was based on helicate Lu^III^32_3_, assembled by combining diamagnetic Lu^III^ with three equivalents of a 2,6-pyridinedicarboxamide (pdc) ligand 32 (Fig. 11a).^20^ The use of naphthol groups increases stabilisation of the complex through inter-ligand π–π interactions and orients the alkene end groups for closure of the tangle.^69^ Connecting each of the three pairs of terminal alkenes by RCM afforded racemic trefoil knot Lu^III^33. When point-chirality was introduced in the form of methyl stereocentres adjacent to the pdc binding sites (34), a trefoil knot of single handedness Λ-Lu^III^35 was formed (*i.e.* the helical arrangement of the ligands around the metal centre is stereocontrolled; Fig. 11b).^70^ Unlike (Δ/Λ)-Zn^II^_3_17_3_, chiral self-sorting of individual enantiometic building blocks did not occur. However, in later work^27^ featuring several chiral pdc units connected within the same strand, self-sorting does occur because of the strain and steric clashes involved in wrapping a multidentate strand around a single metal ion in order to coordinate to several sites at once.

### 2.5 Chaperone-assisted folding and entanglement of single strands

Knotting in polymer chains and proteins occurs by the folding and entanglement of a single strand about itself. This is more reminiscent of how knots are tied in the macroscopic world than the multicomponent self-assembly strategies discussed in this Review so far (although the forces used to drive strand entanglement differ at different length scales).^71^ Tying a knot in a preformed strand simplifies covalent capture of the entanglement: only two reactive ends need to be positioned, incorrect intramolecular connections cannot occur, and only intermolecular reactions (oligomerisation) need to be avoided. However, encoding sufficient structural information to control the number, stereochemistry and sequence of crossings, while also restricting the conformations the strand can adopt, is much more demanding with a single component. With a look to the future, programming entanglements into sequence-specific polymers formed by solid phase synthesis may prove helpful for single strand folding-and-entangling approaches.

**Fig. 12:**
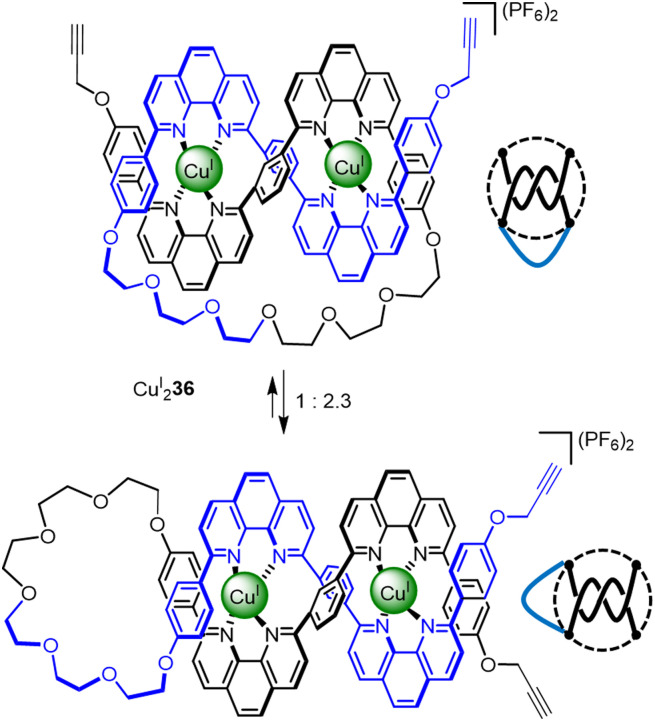
Molecular overhand knot Cu^I^_2_36 and its tangle representation. The complex was generated along with a sequence isomer. The undesired denominator closure in the sequence isomer means that the synthon only generates nugatory crossings which untwist upon demetallation.^72^

An overhand ‘open trefoil’ knot was accessed by Sauvage^72^ (Fig. 12), by performing one numerator closure on a racemic linear helicate containing two Cu^I^ ions (*i.e.* an analogue of Cu^I^_2_2 containing a single closure, Section 2.1) to give Cu^I^_2_36. Unfortunately, Cu^I^_2_36 exists as a dynamic mixture of two conformers, featuring either a numerator or denominator closure. Nevertheless, dimerisation of the impure overhand knot by Glaser coupling gave a mixture of granny and square composite knots (as well as trefoil knot and unknot macrocycle side products), derived from the connection of tangles of the same and opposite handedness, respectively.

Vögtle's serendipitous discovery of hydrogen bonded trefoil knot synthesis (Fig. 2b) also proved to be amenable to a single strand folding approach.^73^ Trefoil knots were prepared in up to 14% yield from a preformed decaamide thread, containing the required six bisphenol Z moieties, and various pyridine dicarboxylic acid dichlorides. In these cases, the overhand knot conformation was not directly observed and yields of trefoil knot were modest, presumably due to the presence of different conformers of the open strand.

**Fig. 13:**
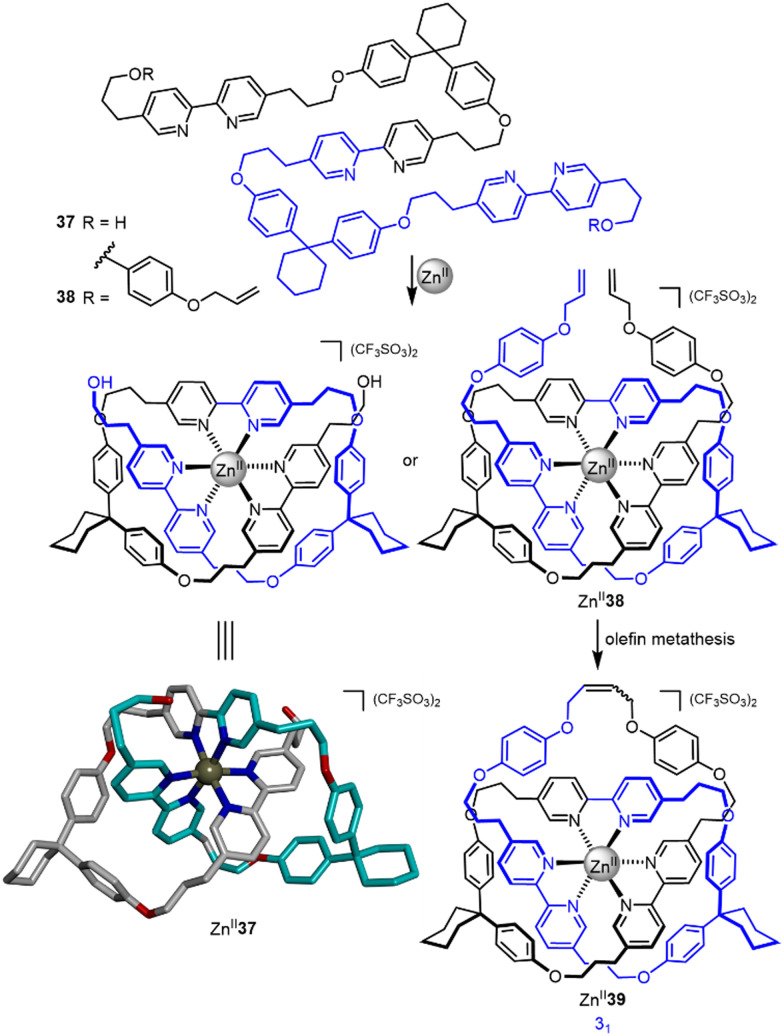
Synthesis of overhand knot Zn^II^_3_37 and trefoil knot Zn^II^_3_39 by folding of a single ligand strand around a Zn^II^ ion.^75,76^

The potential of a single octahedral metal ion to template strand entanglement was postulated by Sokolov as early as 1973,^74^ with the strategy experimentally realized by Hunter and co-workers in 2001^75^ (Fig. 13). A single Zn^II^ ion was used to template the folding of tris-bipyridine strand 37, forming overhand knot Zn^II^_3_37. The bisphenol turn units stabilise the entangled structure by π stacking with the bipyridine groups. A later report detailed closure of Zn^II^_3_37 by bis-esterification and of Zn^II^_3_38 by a single olefin metathesis reaction, the latter affording trefoil knot Zn^II^_3_39 in 68% yield.^76^

**Fig. 14:**
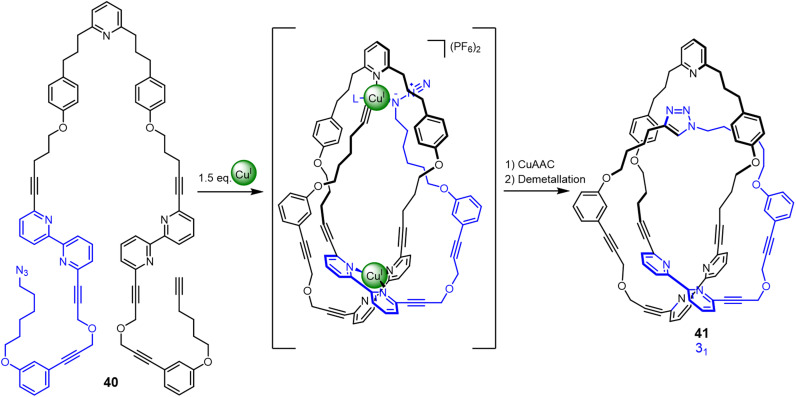
Synthesis of trefoil knot 41 by kinetically controlled active metal template synthesis.^78^

Active template synthesis^77^ uses both the coordination geometry of metal ions to organise (template) building blocks and the catalytic properties of the metal ions to promote covalent capture of an interlocked or entwined product. Unlike many ‘passive template’ syntheses, active template synthesis occurs under kinetic control. Active metal template synthesis has been used to accelerate covalent bond formation through a transiently formed loop to generate a trefoil knot (Fig. 14).^78^ Strand 40 contains three binding sites for two Cu^I^ ions. One tetrahedral Cu^I^ ion forms a crossing point by binding to the two bipyridine groups in the strand, generating the loop. Other Cu^I^ ions catalyse connection of the azide and alkyne groups in a CuAAC (copper-catalysed azide–alkyne cycloaddition) reaction through the resulting cavity to close trefoil knot 41 in 24% yield. Entangled molecular strands formed under kinetic control more closely mimic aspects of the stochastic knotting of synthetic polymer strands and biopolymers.^79^

**Fig. 15:**
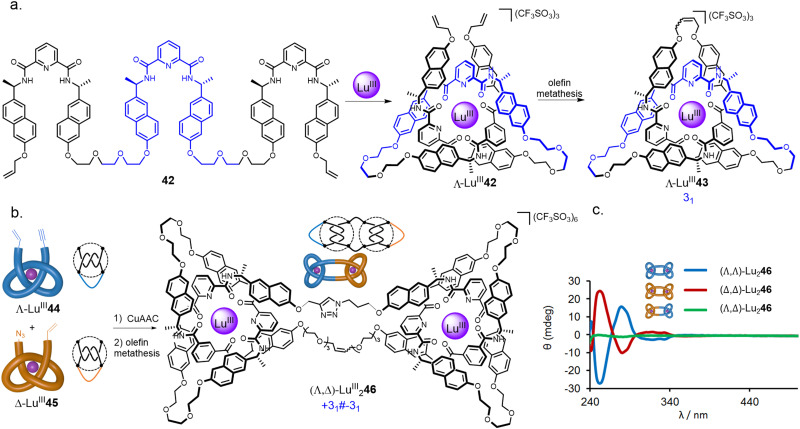
(a) Synthesis of trefoil knot Λ-Ln^III^43 by folding a single ligand around a lanthanide ion.^80^ (b) Stereoselective synthesis of composite knots with tangle representations, here represented by square knot synthesis.^82^ (c) CD spectra of granny and square knots Lu^III^_2_46.^82^ Reproduced from ref. 82 through a CC-BY license.

In addition to the use of transition metal templates, folding and entwining about lanthanide ion templates (see Section 2.4) has emerged as an effective route to molecular knotting.^20^ A folding approach was developed by covalently connecting three homochiral 2,6-pyridinedicarboxamide ligands to form strand 42 (Fig. 15a).^80^ In the presence of Lu^III^ ions, this strand quantitatively folds into enantiopure overhand knot Λ-Lu^III^42. In contrast to the approach using multiple achiral building blocks shown in Fig. 11, a single topological enantiomer of trefoil knot Λ-Ln^III^43 was obtained in 90% yield after the olefin metathesis mediated closure.

An advantage of the folding approach over multicomponent self-assembly is the ability to programme functionality into the knotted strand at specific positions, much like engineering protein secondary structure into peptide sequences.^81^ By introducing different functional groups at the termini of an overhand knot, the stepwise dimerization of lanthanide overhand knots provides composite knots in a stereoselective manner (Fig. 15b).^82^ Connecting bifunctional overhand knot building blocks Lu^III^44 and Lu^III^45 (obtained by modifying 42) of the same handedness gives either enantiomer of granny knot (Λ,Λ)/(Δ,Δ)-Lu^III^_2_46. Alternatively, combining overhand knots of opposite handedness gives a pseudo-meso square knot (Λ,Δ)-Lu^III^_2_46.

The composite molecular knot isomers display marked differences in chiral expression: both enantiomers of granny knot (Λ,Λ)-Lu^III^_2_46 and (Δ,Δ)-Lu^III^_2_46 give pronounced Cotton effects, whilst square knot (Λ,Δ)-Lu^III^_2_46 displays almost no chiral response (Fig. 15c). The very small CD signal in (Λ,Δ)-Lu^III^_2_46 is a consequence of the Λ- and Δ-tangles in the molecule being structurally different in the triazole linker region, preventing the molecule from having a perfect mirror plane.

**Fig. 16:**
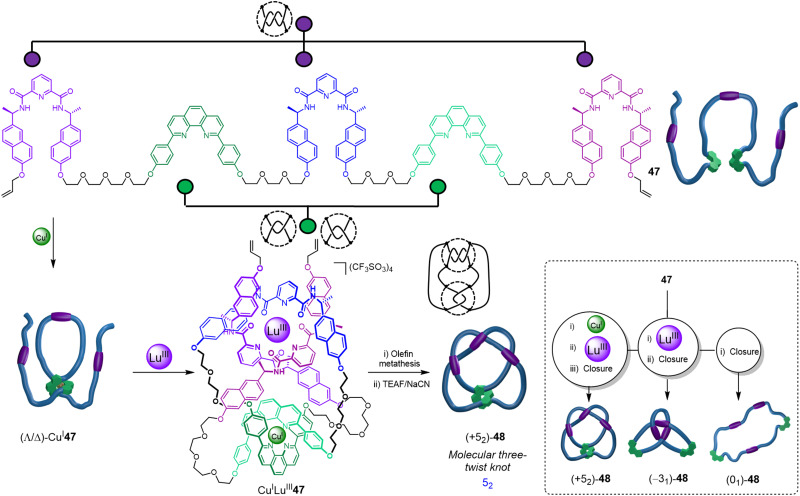
Synthesis of a molecular 5_2_ three-twist knot *via* stepwise folding around Cu^I^ and Lu^III^ templates, followed by covalent capture and demetallation. Tangles are shown next to crossing regions. Inset: A single ligand 47 forms three topoisomers: 5_2_ knot (+5_2_)-48, trefoil knot (−3_1_)-48 and unknot (0_1_)-48.^21^

Recently, the folding-and-entwining approach was used to generate a low symmetry higher-order prime knot, the 5_2_ three-twist knot (Fig. 16).^21^ This was achieved by programming orthogonal coordination units for two different metal ions into the same strand. Pentatopic ligand 47 contains three alternating homochiral 2,6-pyridinedicarboxamide units (to bind to Lu^III^) interspersed with two 1,10-diphenyl phenanthroline sites (to bind to Cu^I^). To access knot (+5_2_)-48, Cu^I^ was first added to generate the first two crossing points by forming ‘clasp’ complex (Λ/Δ)-Cu^I^47. Next, Lu^III^ was added to produce Cu^I^Lu^III^47, generating three further crossing points. As the 2,6-pyridinedicarboxamide units bind to Lu^III^ with a specific helical handedness, the stereochemistry of the Cu^I^ clasp is dictated by the mechanical constraints on the ligand strand: the point chirality that enforces the helical chirality of the [+3] tangle also ultimately directs the handedness of the [−2] tangle. The resulting open knot Cu^I^Lu^III^-47 was closed by olefin metathesis, giving (+5_2_)-48 after demetallation.

The folding-and-entwining strategy allows different knots to be tied in the same molecular strand. Topoisomeric trefoil knot (−3_1_)-48 was obtained when only Lu^III^ coordination was used with 47, whilst unknot (0_1_)-48 resulted if no metal ions were added prior to macrocyclisation. The strand folding showed pathway dependence (*i.e.* is under kinetic not thermodynamic control): if instead the Lu^III^ is added first to 47, the open 5_2_ knot does not form upon addition of Cu^I^. Being able to tie different knots in a molecular strand should aid understanding of the fundamental influence of different knot topologies on chemical and physical properties.

### 2.6. Vernier template knot synthesis

**Fig. 17:**
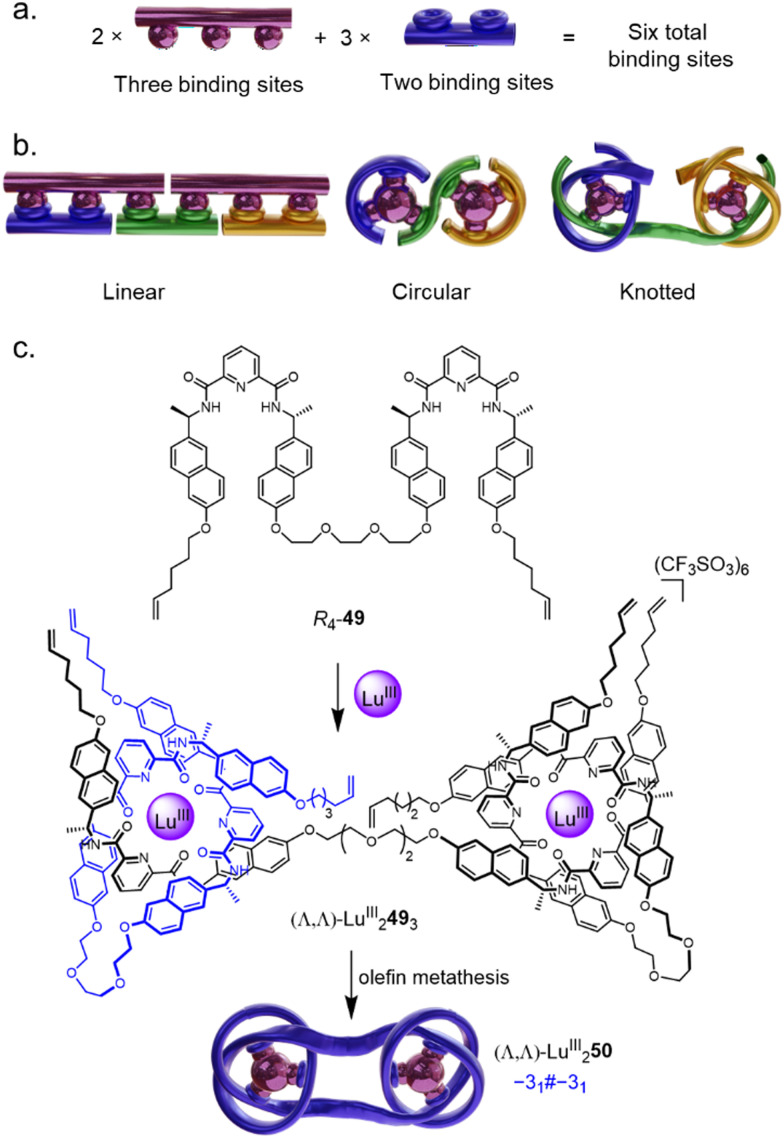
(a) The Vernier template approach to increase the complexity of a system by targeted coordinative mismatch.^83^ (b) The Vernier template approach as applied to linear, circular, and knotted systems in a 2 : 3 ratio.^84^ (c) Synthesis of granny knot (Λ,Λ)-Lu^III^_2_50 by Vernier template synthesis.^27^

The rapid assembly of large and complex composite knots *via* Vernier template synthesis was also recently introduced.^27^ Vernier assemblies rely on a mismatch between the number of binding sites on two components with complementary recognition elements.^83^ The result is a Vernier complex with the lowest common multiple of binding sites of the two components (Fig. 17a). Vernier template synthesis has previously been used to assemble very large, but topologically trivial, macrocycles (Fig. 17b).^84^ The Vernier concept was adapted to the assembly of molecular knots by complexing ligand strands with two or four tridentate pyridinedicarboxamide (pdc) groups with nine-coordinate lanthanide ions: if, instead of a tritopic ligand such as 42,^80^ ditopic ligand *R*_4_-49 is introduced to Lu^III^, a 3 : 2 ligand/metal complex (Λ,Λ)-Lu^III^_2_49_3_ forms, which can be covalently captured by olefin metathesis to form the six-crossing granny knot (Λ,Λ)-Lu^III^_2_50 (Fig. 17c). Despite the lability of the coordination sphere, complete control of topological chirality is retained.

**Fig. 18:**
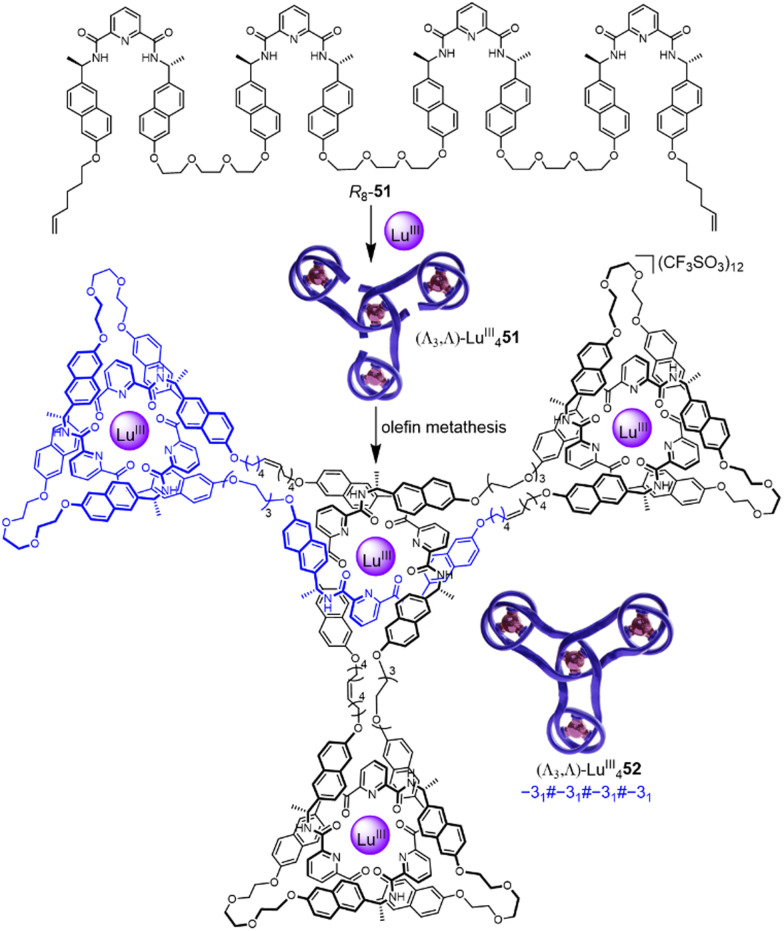
Synthesis of triskelion knot (Λ_3_,Λ)-Lu^III^_4_52 by Vernier template synthesis.^27^

**Fig. 19:**
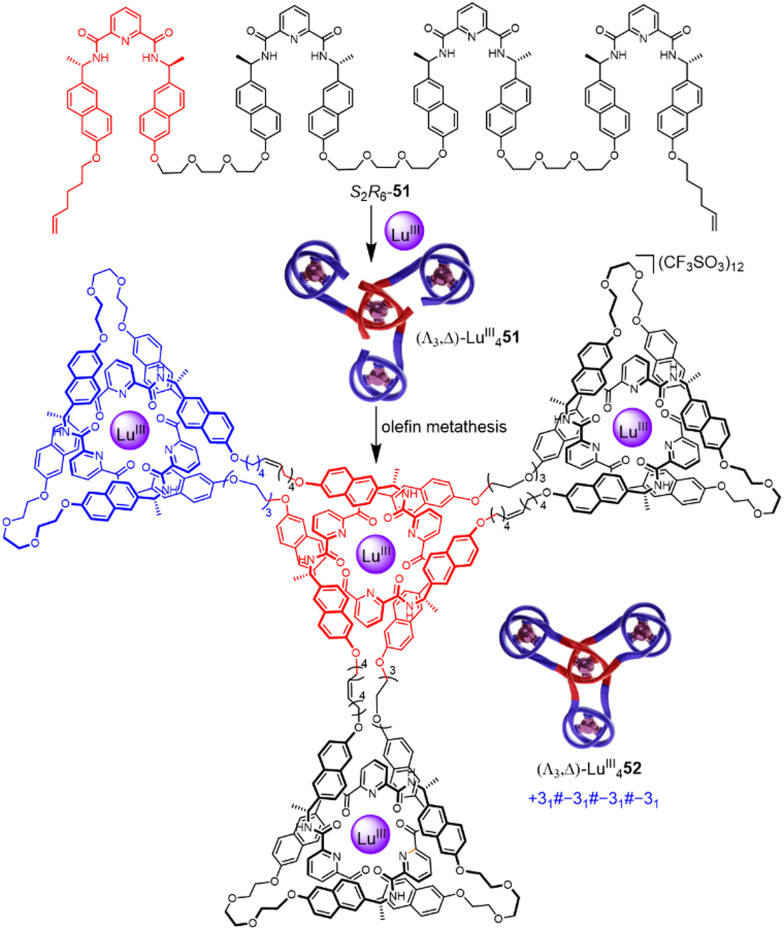
Synthesis of ‘inverted core’ triskelion knot (Λ_3_,Δ)-Lu^III^_4_52 by Vernier template synthesis.^27^

Incorporating four covalently tethered pdc units into strand *R*_8_-51 enables Vernier template synthesis of composite knot (Λ_3_,Λ)-Lu^III^_4_52 featuring 12 crossings—the most topologically complex synthetic molecular knot realised to date (Fig. 18).^27^ The threefold-symmetric triskelion assembly^85^ has 12 alternating crossings and two distinct environments for the coordinated metal ions: three outer trefoil tangles and a central trimeric circular helix. The handedness of the helix could be inverted by programming stereocentres of opposite handedness on just one terminal pdc site of the ligand strand. The resulting ‘inverted core’ triskelion knot (Λ_3_,Δ)-Lu^III^_4_52 is an isomer of triskelion knot (Λ_3_,Λ)-Lu^III^_4_52, and has six alternating and six non-alternating crossings (Fig. 19).^27^

The Vernier template synthesis approach combines aspects of the circular helicate and folding-and-entwining strategies. It facilitates the hierarchical construction of knots and the rapid assembly of large, complex entangled structures (with molecular weights exceeding 8 kDa) from relatively simple building blocks.

### 2.7. Knots and links from interwoven molecular grids

Molecular grids consist of polytopic ligands coordinated to two-dimensional arrays of metal ions. Interest in this class of assembly includes the electronic and magnetic properties of well-defined spatially separated and organised metal ions within a molecular framework.^86^ Almost all of the molecular grids reported to date are racks, *i.e.* consist of stacked layers of ligand strands that are not woven through the plane described by the metal ions. Connecting the ends of adjacent ligands in such grids would only produce unknot macrocycles of various sizes. Interwoven grids, where each ligand strand passes back-and-forth and over-and-under other strands through the plane of metal ions, have the potential to generate strand entanglements. The use of grids as precursors for knots and more extended molecular weaves was first postulated three decades ago by Busch: “*The ultimate aspiration of chemists working on interlocked structures might be to weave molecules as if they were macroscopic threads*”.^22*a*^

**Fig. 20:**
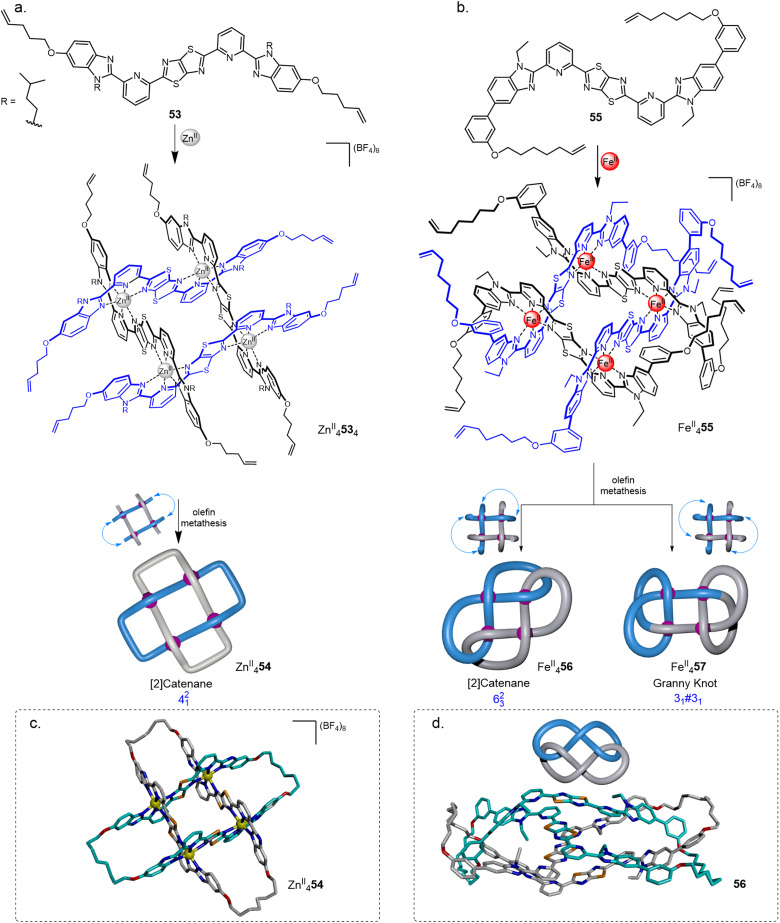
Synthesis of 2 × 2 interwoven molecular grids and their closures by olefin metathesis to generate (a) Solomon link 54;^14^ or (b) 6^2^_3_ link 56 and granny knot 57;^15^ X-ray crystal structures of (c) Zn^II^_4_54 and (d) demetallated link 56.

The use of interwoven grids for the synthesis of molecular knots was realised using ligands containing a central thiazolo[5,4-*d*]thiazole (TTZ) moiety.^87^ The TTZ moiety is flanked by pyridylbenzimidazole units to induce a zig-zag shape in ligand coordination and favour the formation of interwoven grids. Treatment of ligand 53 with M^II^(BF_4_)_2_ (M = Zn, Co, Fe) quantitatively formed 2 × 2 interwoven grids M^II^_4_53_4_.^14^ Solomon link Zn^II^_4_54 was obtained in 72% yield (Fig. 20a and c) by closing the corresponding grid ends using olefin metathesis. Demetallation with Li_2_S or Na_4_EDTA gave the wholly organic doubly interlocked link 54.

Modified ligand 55 and Fe^II^(BF_4_)_2_ gave 2 × 2 interwoven grid Fe^II^_4_55_4_, for which the ligands are forced into a conformation such that adjacent olefin chains are directed to opposite faces of the grid. Suprafacial connections (Fig. 20b) of alternate ligands gave a mixture of a 6^2^_3_ link Fe^II^_4_56 (a twisted [2]catenane) and the isomeric composite granny (+3_1_#+3_1_/−3_1_#−3_1_) knot Fe^II^_4_57.^15^ The isomers were separable using gel permeation chromatography. The X-ray crystal structure of demetallated link 56 (Fig. 20d) revealed an extensive network of intramolecular aromatic stacking.

**Fig. 21:**
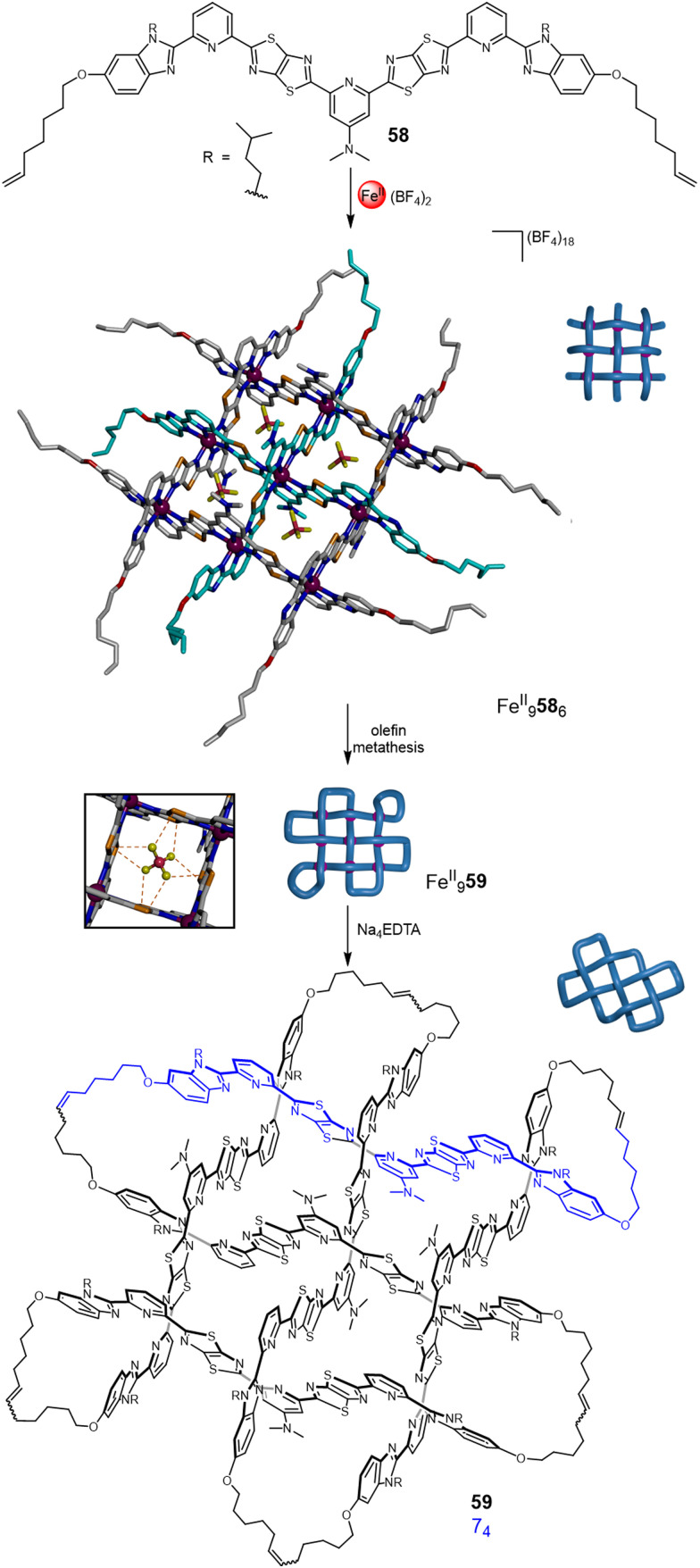
Weaving molecular endless (7_4_) knot 59 by self-assembly of an interwoven 3 × 3 grid.^16^ Inset: The stabilising effect of the BF_4_^−^ anions through B–F⋯π and electrostatic interactions.

The strand connectivities set up by their positioning within interwoven grids is different to that possible with circular or linear helicates, enabling different topologies to be accessed through the use of more than two weft and two warp ligands.^9*b*^ This was demonstrated using a 3 × 3 interwoven grid to template the assembly of an ‘endless’ (7_4_) knot, an iconic knot and symbol common to many cultures and religions (Fig. 21).^16^ The 3 × 3 grid was assembled by treating tritopic ligand 58, featuring two TTZ moieties to generate the over-under-over weave, with Zn(BF_4_)_2_ or Fe(BF_4_)_2_. The BF_4_^−^ anions proved to be crucial for grid assembly, without them it does not form. The X-ray crystal structure of Fe^II^_9_58_6_(BF_4_)_18_ shows BF_4_^−^ anions bound within each of the four square cavities of the complex, stabilising the assembly through B–F⋯π and electrostatic interactions. Joining the ligand termini within the grid by olefin metathesis followed by demetallation with Na_4_EDTA afforded molecular 7_4_ knot 59. A Solomon link and unknot macrocycle are also formed as side-products from other patterns of strand closure on the grid.^16^

### 2.8. Knots from covalent templates

**Fig. 22:**
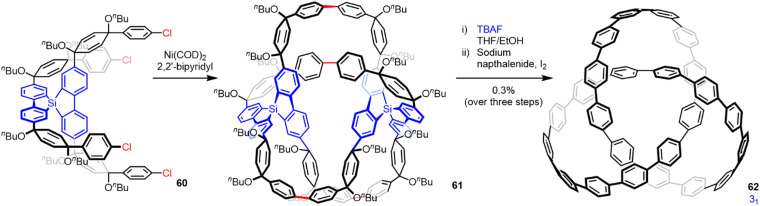
Synthesis of a molecular trefoil knot using covalent templates.^91^

Covalent templates and scaffolds were used in many early attempts at the synthesis of interlocked and entwined molecules.^88^ Schill, one of the pioneers of this approach, was unsuccessful in efforts to synthesize a molecular trefoil knot using a covalent template based on quinone.^89^ The failure was partly due to the lengthy and rather convoluted synthesis necessary because of the synthetic methods available at the time, but removing the template attached to the putative knotted strand through several chemically robust covalent bonds was also problematic.^89^ Despite a number of other attempts over the subsequent five decades,^90^ the first report of a trefoil knot synthesized through a covalent template strategy was disclosed by Itami and co-workers in 2019.^91^ They used this approach in a synthesis of two cycloparaphenylene [2]catenanes and a trefoil knot 62 (Fig. 22). The hydrocarbon trefoil knot was synthesized through the statistical dimerization of 60, containing a tetrahedral arylsilane template to give ‘fused knot’ 61, followed by template removal and oxidation to generate 62 in 0.3% yield. Knot 62 is a remarkable model structure through which the effects of chirality, curvature and topology on entangled carbon nanostructures can be studied.

### 2.9. Recent advances in knot synthesis through hydrophobic assembly

**Fig. 23:**
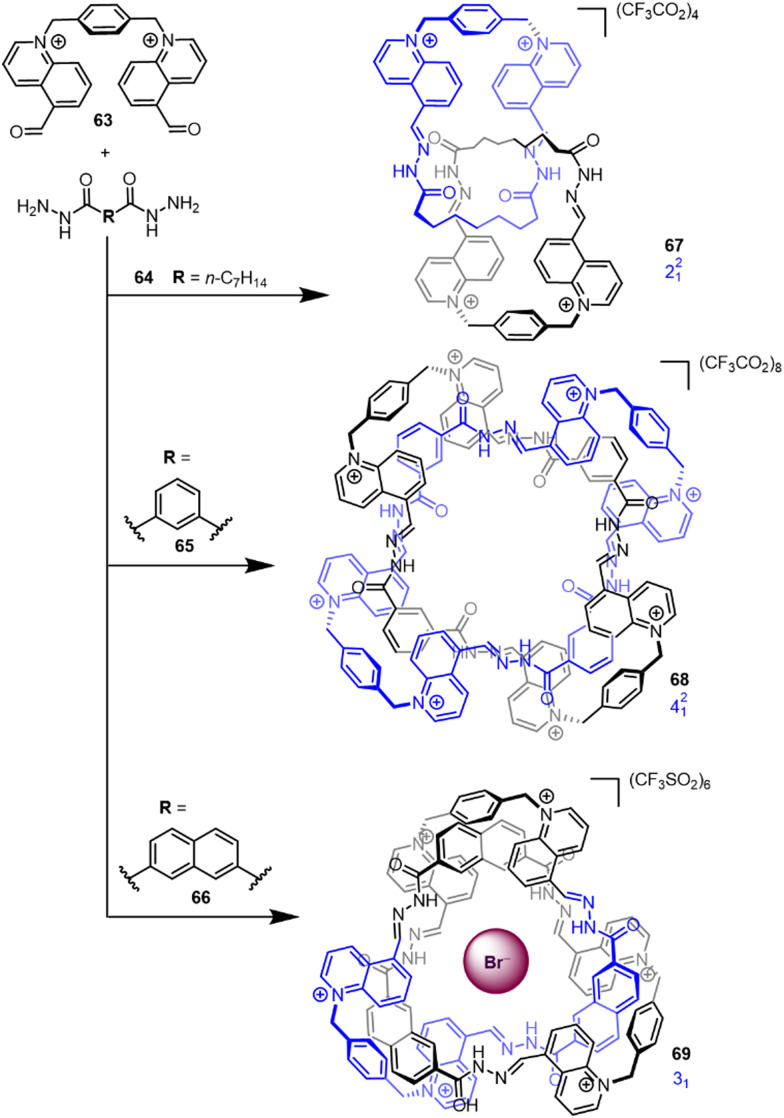
Effect of hydrazide linker R on the obtained topology when using hydrazone formation in water.^18^

Although several notable early knot syntheses utilised solvophobic effects (see Section 2.1), the lack of control over product formation limited its application as a design strategy. However, recently Cougnon has used the hydrophobic effect to access a number of interlocked structures (Fig. 23).^18^ Assemblies form by dynamic hydrazone formation upon mixing of bisquinolinium dialdehyde 63 and dihydrazides 64–66 in water (see Section 2.3.1 for metal template knots by imine bond formation). The topology of the entangled product is dictated by the choice of counter-anion and dihydrazide bridging unit, and could be biased to generate either Hopf link 67, Solomon link 68 or trefoil knot 69 in 70–90% yield. The aldehyde and hydrazide building blocks entangle to minimise the amount of organic molecular surface exposed to water. The conformation of 68 was found to be sensitive to the amount of water present, with even small amounts of moisture inducing a large conformational switch.^19^

### 2.10. Metalla-knots

The syntheses discussed in Sections 2.3–2.7 mostly rely on metal ions as scaffolds around which organic ligands are entangled. Metalla-knots are metal complexes where the metal ions form part of the knotted strand itself and so cannot act as removable templates.† Metalla-knots are often dynamic structures that only exist within a particular range of conditions, sometimes only in solution or only in the solid state. Due to the often dynamic nature of metal–ligand bonding, strand regions may not be mechanically restricted within a particular topology. In that case the effects of entanglement upon structure and properties may be limited. Nevertheless, metalla-knots have been found to form a wide range of topologies that have yet to be realised with wholly organic strands.

A variety of metals have been employed in metalla-knots, including some from the 2nd and 3rd rows of the periodic table.^92^ This is in contrast to metal template molecular knots, which generally use either 1st row transition metals or lanthanides. Certain coordination geometries, such as linear or square planar, are more readily accessed with heavy transition metals. The majority of the ligands used in metalla-knots feature pyridyl-type end groups in metal coordination.

The earliest example of a metalla-knot was reported by Hosseini in 2008 and featured linear coordination between Ag^I^ and quinoline units linked by glycol chains to generate complexes with either a trefoil or figure-eight topology.^93^ Secondary interactions between the metal ions and the glycol linkers were apparent in the X-ray crystal structures of the metalla-knots. Jin has since described a wide variety of metalla-knots based on organometallic dimers and dipyridyl ligands.^94^ In some cases the dynamic coordination behaviour characteristic of metalla-structures has been exploited to produce stimuli responsive interconversion of different species.^95^

**Fig. 24:**
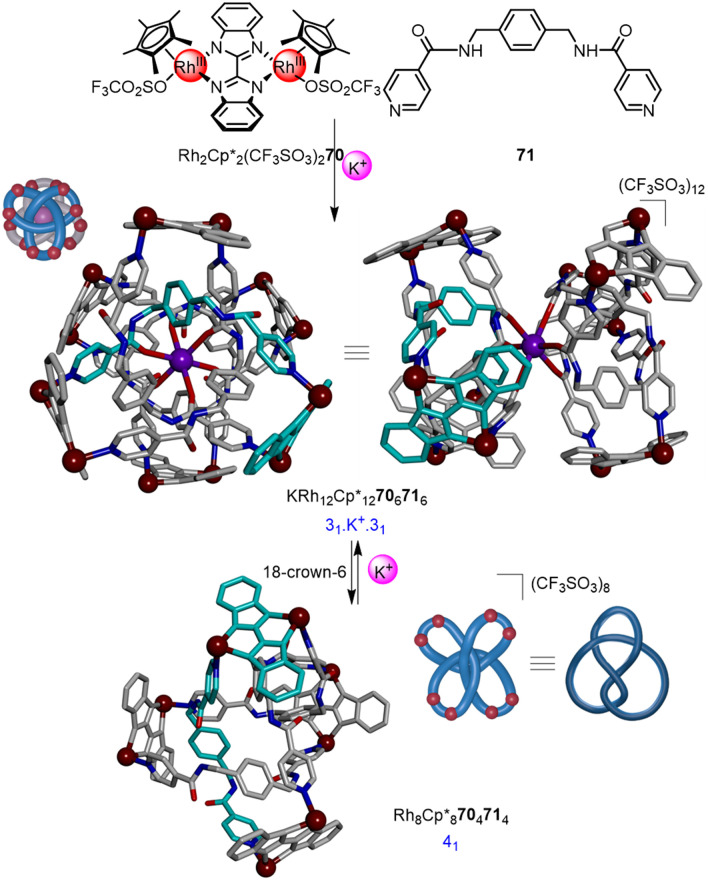
Synthesis of a ‘double-3_1_’ metalla-knot and its interconversion with a 4_1_ metalla-knot mediated by K^+^. Additional K^+^ ion and Cp* ligands are omitted from the X-ray crystal structure representations for clarity.^95*b*^

Optimisation of the component structures—dimeric Rh^III^ or Ir^III^ half-sandwich complexes and bis-pyridyl linkers—has led to metalla-knots and links of remarkable stability.^96^ The majority of metalla-knots reported by Jin are trefoil knots,^94*b*–*d*^ but also include figure-eight knots,^97^ an unsymmetrical trefoil knot that interconverts with a Solomon link,^95*a*^ and a ‘double’ trefoil knot bridged by alkali metal ions.^98^ Particularly noteworthy is the K^+^ mediated interconversion of the double 3_1_ knot KRh_12_Cp*_12_70_6_71_6_ and 4_1_ knot Rh_8_Cp*_8_70_4_71_4_ (Fig. 24), a rare example of dynamic interconversion of molecular entanglements.^95*b*^ Most recently, the Jin group reported the selective synthesis of either an 8_18_ knot, Borromean rings or unknot metallacycle from subtly different ligands, highlighting the fundamental role of π–π interactions in the assembly of such systems.^99^

**Fig. 25:**
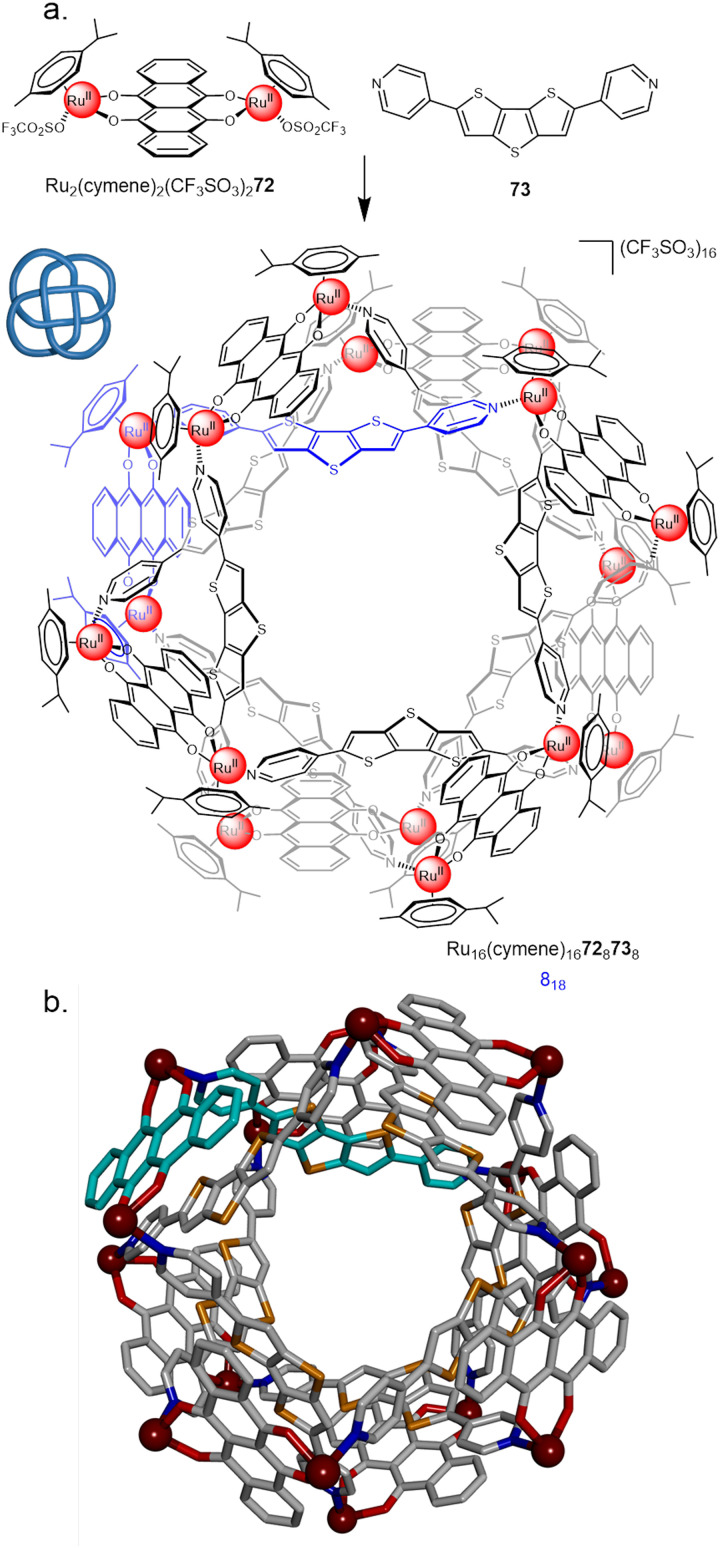
(a) Synthesis of 8_18_ metalla-knot Ru_16_(*p*-cymene)_16_72_8_73_8_. (b) X-ray crystal structure of the 8_18_ metalla-knot with *p*-cymene ligands omitted for clarity.^100^

In 2018 Chi and co-workers employed Ru^II^ dimer Ru_2_(*p*-cymene)_2_OTf_2_72 in the synthesis of an 8_18_ metalla-knot (Fig. 25).^100^ Combining the dimer with an equimolar ratio of dipyridyl ligand 73 gave [8+8] assembly Ru_16_(*p*-cymene)_16_72_8_73_8_ in 74% yield. Recently, the Chi group also reported a 6^3^_1_ link, accessed by combining the same Ru^II^ dimer with a bipyridyl linker with a slightly larger bend angle.^101^ This sensitivity to ligand geometry highlights some of the advantages and limitations of metalla-knots: small changes can afford a diverse range of topologies, but structural designs are therefore difficult to confidently predict in advance.

**Fig. 26:**
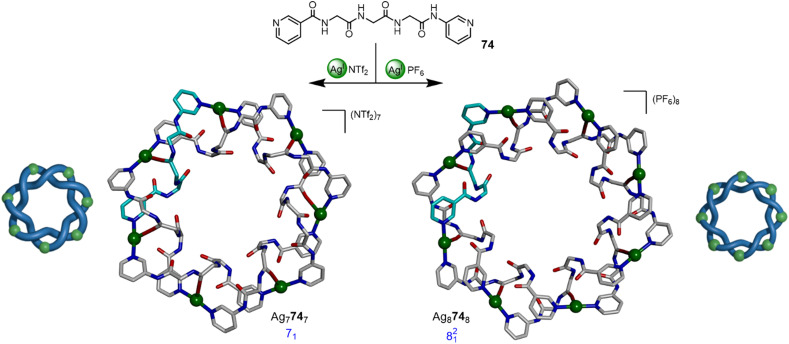
Anion-mediated synthesis of 7_1_ metalla-knot Ag_7_74_7_ or 8^2^_1_ metalla-link Ag_8_74_8_ by coordination of Ag^I^ ions to pyridyl-capped polypeptide ligand 74.^103^

Fujita and coworkers have reported a number of higher-order metalla-entanglements based on the coordination of Ag^I^ ions to pyridyl-capped polypeptide ligands.^102–104^ Examples include a series of topoisomeric [4]catenanes^102^ and the divergent synthesis of 7_1_ and 8^2^_1_ metalla-knots (Fig. 26).^103*a*^ The combination of a flexible triglycine ligand 74 with AgNTf_2_ in a 1 : 1 ratio affords 7_1_ metalla-knot Ag_7_74_7_, whilst using AgPF_6_ gives 8^2^_1_ metalla-link Ag_8_74_8_. Most recently the group reported the synthesis of 9_1_ and 10^2^_1_ metalla-knots.^103*b*^ The higher-order topologies were accessed by changing the steric bulk of the ligand sidechains and varying solvent. The X-ray crystal structures show that the self-assembly relies on counter-ion binding, hydrogen bonding, π–π interactions and Ag–O coordination, demonstrating the complex structural balance necessary for short peptide sequences to promote entanglements through complementary inter-strand non-covalent interactions.

A route to enantiopure circular helicates *via* a chiral-at-metal Ir^III^ motif has also been introduced.^105^ The kinetic inertness of the Ir^III^–C bond means that the enantiospecific synthesis of a heterometallic Ir_2_Zn_4_ Star of David link could provide a suitable route to equally dynamically robust metalla-knots.

### 2.11. Molecular weaving

Molecularly woven materials share many of the same design principles and structural considerations as molecular knots.^106^ The goal of nanoscale weaving was discussed following the early use of metal templates in catenane synthesis,^22*a*,107^ but only in recent years have the first molecularly woven materials been prepared. The presence of long-range order (*i.e.* orderly molecular entanglements) differentiates woven materials from the random strand knots and tangles that occur generally with polymers of sufficient length and flexibility.^108^ In contrast to macroscopic weaving, in which pre-formed threads are passed over and under each other to build up the material,^109^ molecularly woven fabrics can be prepared from pre-formed tangles or reticular chemistry. Rather than connecting crossings intramolecularly to form a molecular knot or link, intermolecular connections can form extended woven networks.

**Fig. 27:**
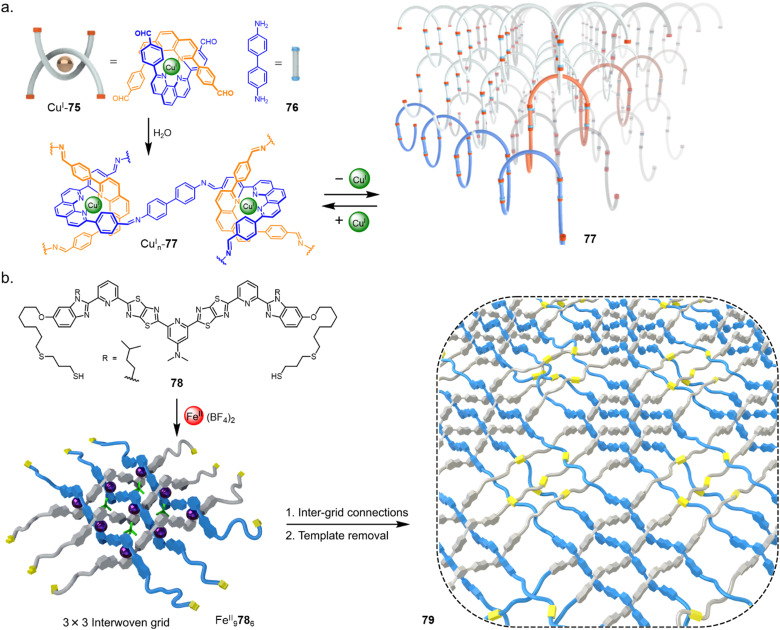
(a) Synthesis of a 3D interwoven covalent organic framework (COF) by reticular chemistry.^24^ (b) Synthesis of a 2D interwoven fabric by tessellation of discrete layers of molecular grids.^23^

In 2016 Yaghi and coworkers reported a material molecularly woven in three-dimensions through reticular chemistry (Fig. 27a).^24^ Bisphenanthroline Cu(i) complex Cu^I^75 positions the four aldehyde groups in a tetrahedral geometry. Subsequent condensation of Cu^I^75 with diamine 76 forms 3D interwoven COF Cu^I^_*n*_77. Demetallation with KCN yielded COF 77 with only 3–8% of the Cu^I^ ions that hold the strands in fixed registry remaining. The result was a material with a tenfold increase in elasticity.

A 2D supramolecular triaxial Kagome weave has been reported by the Wennemers group, featuring woven strands with intermolecular π–π interactions between perylene monoamide groups. Although the non-covalent connectivity may enable the strands to pass through each other in this material, the relative robustness and uniform pore structure augurs well for separation and storage-type properties.^25^ Mayor has used a surface-mounted MOF template approach to synthesise a single layer 2D woven polymer network. However, the structure did not prove sufficiently robust to survive removal of the template.^26^

The 2D molecular weaving of polymer strands has been achieved by tessellation of a preformed 3 × 3 grid (Fig. 27b).^23^ Ligand 78, derived from 58 used to form a 7_4_ knot (see Section 2.7),^16^ is appended with thiol end groups. Following formation of interwoven 3 × 3 grid Fe^II^_9_78_6_, crosslinking by disulfide formation was carried out in air to form a layered molecularly woven material Fe^II^_*n*_79. Demetalation with KCN afforded a metal-free organic woven polymer 79 in nanosheet layers of uniform 4 nm thickness which possessed long-range order. Notable property differences were found between the woven 2D polymer sheets and non-woven 1D strands of the same polymer, including thermal stability, stiffness and ion permeability. The ability to weave polymer chains in two-dimensions—forming molecularly woven fabrics—marks the intersection of three major research fields: polymer science, two-dimensional materials and molecular nanotopology.^106^

## 3. Effects on properties of molecular entanglements

### 3.1. Property changes caused by strand entanglement

Entangling strands at any length scale can have a significant effect on properties. For example, knotting a rope reduces its tensile strength, because an applied stretching force becomes unevenly distributed across the fibres at the entry to the knotted section.^110,111^ In such a case the reduction in strength varies with the knot topology.^112^ Similar effects have been observed when stretching knotted macromolecules, such as actin filaments and DNA.^113^ Simulations on a polyethylene chain indicate that the tighter the knot (*i.e.* the shorter the average distance per crossing) the more severe the strain imposed at the knot apex becomes.^114,115^ The weakening of the molecular strand is caused by distortion and weakening of the covalent bonds at or close to the knot apex.

Whether a strand region crosses over or under another introduces a new stereochemical element and most knots are consequently chiral.‡‡An exception is amphichiral knots, such as the figure-eight (4_1_) knot. Whilst the reduced representation of this knot cannot be depicted in an achiral form, the knotted strand can, in principle (of course it may be possible to prevent this sterically in a real molecule!), be rearranged into either mirror image of the representation. Accordingly, the 4_1_ knot is topologically achiral. Such ‘topological chirality’ leads to enhanced Cotton effects of knots compared to their unknot topoisomers or individual components, as a result of the restriction of conformational freedom within the topologically chiral environment defined by the knotted closed loop.^116^ Entangling a strand often results in a change in the diffusion constant determined by DOSY NMR spectroscopy^21^ or shorter drift times measured by ion-mobility mass spectrometry.^78^ Furthermore, the burying of solvophobic functional groups within knots, sometimes utilised as a driving force for entanglement, can cause changes in molecular polarity and solubility.^13,18^

Approximately 1% of proteins in the Protein Data Bank are knotted,^9*c*,71,117^ which is substantially lower than would be expected for stochastic entangling of such long and flexible chains.^118^ The reasons for such a low prevalence of knots in proteins are not fully understood, but the slow kinetics of entangling a polypeptide chain seems to disfavour knotting. Several functionally essential proteins such as SPOUT methyltransferases (3_1_) and ubiquitin hydrolase (5_2_) contain knots that are highly conserved across the protein families.^71^ There is also evidence that knotted conformations can bring hydrophobic and hydrophilic parts of proteins closer together, a useful feature for enzyme active sites.^117^ It may not be coincidence that >80% of the known knotted proteins are enzymes. Another prevalent hypothesis is that knots increase the kinetic stability of proteins because the entangled region suppresses degradation by preventing entry of the protein into the proteasome.^119^ However, there is still ongoing debate whether knotting in proteins has been selected by evolutionary pressure, or if their occurrence remains relatively unimportant for function.

In contrast to proteins, DNA undergoes stochastic knotting under biotic conditions.^120^ Knotted and supercoiled DNA strands are unable to undergo transcription, replication or recombination, and therefore nature uses topoisomerase enzymes to mediate knotting and supercoiling.^121^ Failure to remove knots leads to cellular death.^9*c*,122^ Spontaneous knotting has been implicated as a potential problem in DNA nanopore sequencing, as such pores may be unable to allow passage of knotted DNA strands.^123^

### 3.2. Effects of knot tightness

Tying a knot restricts the conformational space a strand can sample, a process that has an entropic cost that depends on the minimal knotted length.^124^ Hence, the tightness of a molecular knot is a useful metric for understanding some of the potential effects on properties. The backbone crossing ratio (BCR) is a measure of how tight a molecular knot is tied based purely on chain length, rather than chemical functionality.^125^ It is calculated by dividing the number of atoms in the shortest path along the backbone of the knotted strand by the total number of crossings (*i.e.* atoms per crossing). The BCR lies between 27–33 for most molecular knots synthesized to date (Table 1). This corresponds to an average strand length per crossing of approximately 3 nm, suggesting there may be a ‘sweet spot’ in terms of the entropic costs of fixing the crossings *versus* the geometric restrictions needed to template the entanglement.

**Table 1:** BCR ratios of selected molecular knots

Knot type	Crossing #	Knot	Backbone *n*	BCR	Ref.
8_19_	8	26	192	24	59
3_1_	3	41	76	25	78
3_1_	3	39	80	27	76
3_1_	3	69	81	27	18*a*
3_1_	3	2	84	28	12*a*
3_1_	3	5	84	28	13
4_1_	4	6	112	28	17
5_2_	5	(+5_2_)-48	143	29	21
3_1_	3	43	89	30	80
3_1_	3	3	96	32	31
5_1_	5	9	160	32	43
3_1_#3_1_#3_1_#3_1_	12	52	378	32	27
3_1_#3_1_	6	46	199	33	82
3_1_#3_1_#3_1_	9	23	324	36	58
7_4_	7	59	258	37	16

**Fig. 28:**
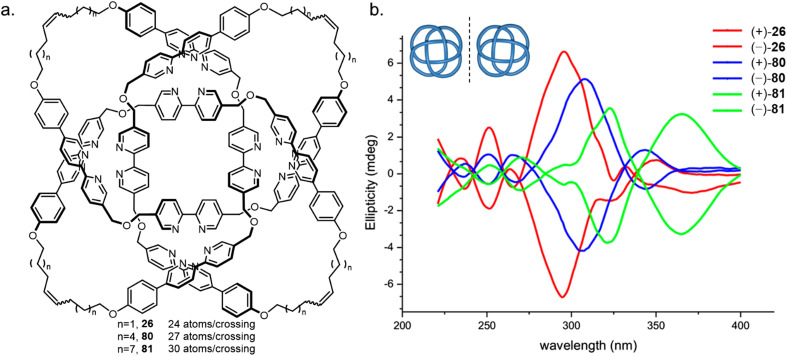
Variation in molecular knot tightness. (a) Three 8_19_ knots 26, 80, 81 with varying length alkyl chains. (b) CD spectral stack plot showing the effect of knot tightness on the expression of chirality.^126^ Reproduced from ref. 126 with permission from the National Academy of Sciences, copyright 2019.

Knots with high BCRs have either been synthesised with nugatory crossings (7_4_ knot 59, BCR 37) or required rigid linkers (3_1_#3_1_#3_1_ knot 23, BCR 36). The theoretical minimum BCR possible for a polyethylene chain knot is proposed to be 15, whilst the lowest value achieved to date is 24, for 8_19_ knot 26.^59^ This knot was used as the basis of a study into the effects of strand tightness.^126^ Analogous knots with a BCR of 27 (80) and 30 (81) were formed by increasing the length of the flexible alkyl chain linker (Fig. 28). The three metallated knots were produced in similar yields and exhibited near-identical ^1^H NMR spectra. However, demetallation of 81 using hydroxide is faster than for 26, suggesting that the metal ions are more accessible within a looser knot. Weaker Cotton effects and red shifting in the CD spectra, as well as smaller diastereotopic splitting of the dibenzyl ether protons in the ^1^H NMR spectrum for 80 and 81 also corroborate that the bipyridine units are held in a less well-defined helical arrangement. Finally, knot 26 was found to have the lowest fragmentation energy by tandem mass spectrometry analysis, consistent with tighter knots being significantly more strained. The experimental findings were supported by molecular dynamics and bond order simulations. The effects of linker length and composition on the tightness of overhand knots have also been explored, identifying a ‘pinching effect’ of a short peptide sequence as the cause of conformational strain effects observed by ^1^H NMR and CD spectroscopy.^127^

Similar conclusions were drawn by Kumpulainen and Cougnon in a study of links 67, 68 and trefoil knot 69, along with an unknot macrocycle.^128^ Flexible Hopf link 67 and the unknot macrocycle had photophysical properties similar to a monomeric model compound, but Solomon link 68 and trefoil knot 69 saw substantial red shifts of the absorption band maxima and lowered p*K*_a_ values (from ∼11 to ∼9) for the hydrazone protons. This was attributed to the higher packing density resulting in a significant reduction in solvent-accessible surface area and the favouring of π–π interactions. From this study, and that of Fig. 28, it is also apparent that some effects of knotting such as chirality expression (with variations depending on backbone rigidity) may start to drop with BCRs over ∼30 (Table 1).

**Fig. 29:**
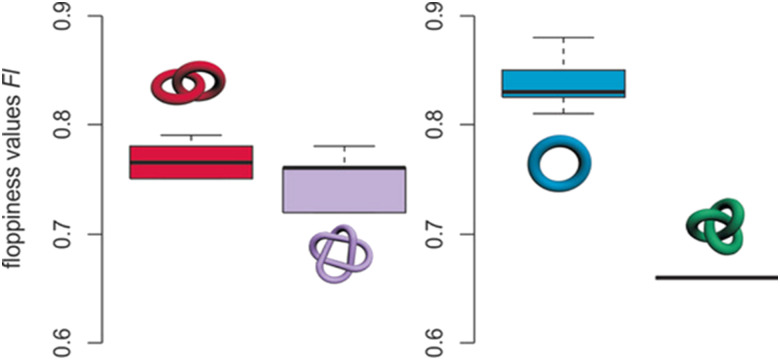
Floppiness values Fl for Hopf links (six, left, red), Solomon links (two, left, violet), macrocycles (eight, right, blue), and a trefoil knot (right, green). The box shows the 50% of the range of floppiness values, thick line shows median. Reproduced with permission from Wiley-VCH.^129^

Schalley and co-workers have found that the time it takes a strand to untangle under collision induced dispersion (CID) conditions in travelling wave ion mobility mass spectrometers (TWIMS) varies predictably with topology and tightness.^129^ They used this to screen complex topological mixtures and to distinguish different species based on their size, shape and packing. The authors defined a so-called ‘floppiness factor’, by dividing the arrival time of the parent molecule by that of the least entangled fragment, corrected for mass and charge (Fig. 29). If the parameter proves to be a reliable predictor in other systems, it could potentially be used to forecast the degree of entanglement in knots.

### 3.3. Dynamics and conformational switching of molecular knots

**Fig. 30:**
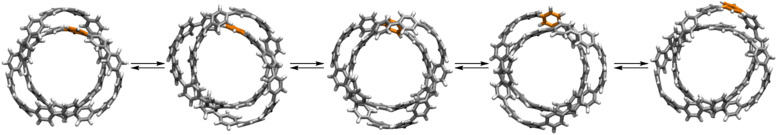
Entanglement dynamics of all-benzene trefoil knot 62 as illustrated with snapshots from DFTB-MD simulations.^91^

The dynamic behaviour of knotted biopolymers such as DNA has been the subject of many experimental and theoretical studies.^35*b*,130^ Knot migration along polymer strands is dominated by reptation, the ‘snake-like’ motion of a chain in an entangled environment.^131^ A striking example of reptation occurs with the all-benzene trefoil knot 62.^91^ Simulations of the NMR spectra indicated that the static knot should exhibit 15 unique proton environments, but due to the rapid dynamic motion the ^1^H NMR spectrum of the knot consists of a single coalesced peak at 7.1 ppm, even at −95 °C. The dynamics of 62 were simulated by density-functional tight-binding with molecular dynamics methods (DFTB-MD; Fig. 30). The motion alters the positions of individual benzene rings from the undercrossing to the overcrossing regions of the knot.

The accessible conformational space for mechanically entangled molecules such as rotaxanes and knots is often greater than might be predicted empirically.^132^ For example, despite several attempts to do so it has not yet proved possible to construct a kinetically inert overhand knot held in place solely through bulky groups on the strand ends.^73,133^

**Fig. 31:**
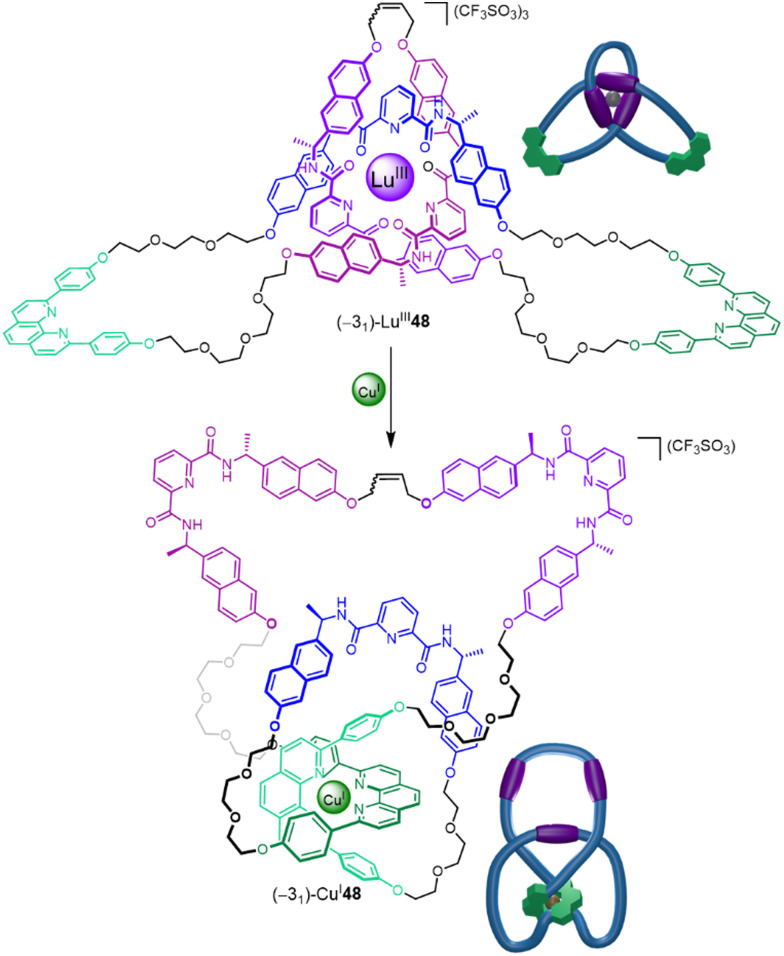
Binding different metal ions changes the position of an entanglement within molecular knot (−3_1_)-48.^21^

Being able to control and utilize the dynamics of entanglements is an important target for exploiting the special characteristics of molecular knotting. Trefoil knot (−3_1_)-48 contains orthogonal coordination sites for both Lu^III^ and Cu^I^ (Fig. 31).^21^ Coordination to a Lu^III^ ion occurs through the three pyridinedicarboxamide groups to give (−3_1_)-Lu^III^48, which adopts a pseudo-*C*_3_ symmetric conformation with a writhe of 3 (Fig. 31 top). Treatment of (−3_1_)-Lu^III^48 with Cu^I^ salts displaces the Lu^III^ ion to afford (−3_1_)-Cu^I^48.^21^ In this complex the knot coordinates to the Cu^I^ through the two phenanthroline groups forcing the ligand strand to adopt a less symmetrical conformation with a writhe of 4 (Fig. 31 bottom). The ability to move the entanglement to different parts of a strand and change writhe may prove useful for introducing strain at given positions, allowing specific bonds to be distorted and weakened on demand.

## 4. Functional molecular knots

### 4.1. Host–guest chemistry

**Fig. 32:**
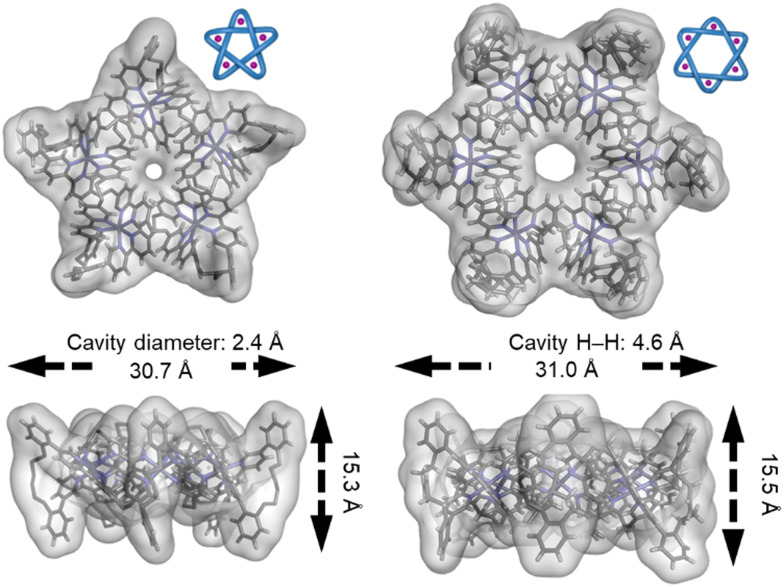
The central cavities of pentafoil knot Fe^II^_5_20 and Star of David catenane Fe^II^_6_21, visualized by overlaying the crystal structures with the solvent-accessible surfaces.^55,56,139^ Reworked from ref. 139 by a CC-BY license.

High anion binding affinities have been found across several different classes of molecular torus knots.^18*a*,134,135^ The tight three-dimensional framework imposed upon a knotted strand can generate cavities capable of forming strong and selective host-guest interactions. A number of X-ray crystal structures of metallated knots show cavity-bound anions, stabilized by positively charged metal ions positioned around the cavity by the entangled ligand strand (Fig. 32). The anions are bound by a combination of electrostatic and [C–H⋯anion] interactions.

A comparison of halide binding within the central cavity of a series of knots and links assembled from circular helicates has been determined for pentafoil knot Fe^II^_5_9, Solomon link Fe^II^_4_11, Star of David Fe^II^_6_21 and 8_19_ knot Fe^II^_4_26.^134^ Pentafoil knot Fe^II^_5_9 has binding affinities for Cl^−^ and Br^−^ of *K* ≈ 10^10^ M^−1^ in MeCN, comparable in strength to the affinity of halide anions for Ag^I^. Anion binding by positively charged metal-knot complexes has also been observed with Trabolsi's Zn^II^–imine trefoil knot, which displays moderate affinity for Br^−^, I^−^, N_3_^−^, SCN^−^ and NO_3_^−^ (*K* ≈ 10^2^–10^4^ M^−1^) in D_2_O.^135^ In this case a 2 : 1 anion : knot binding stoichiometry was always observed, along with positive cooperativity in most cases (*K*_2_/*K*_1_ up to 22). This suggests that the first anion preorganizes the host cavity by restricting the entangled ligand to a specific conformation.

Cougnon's hydrazone trefoil knot 69 and Solomon link 68 display affinity (*K* ≈ 10^4^ M^−1^) towards halides in water.^18*a*^ Two anions accept six preorganized intra-cavity N–H hydrogen bonds from the hydrazones. Stimuli-responsive guest capture and release can be affected by changing the pH as deprotonation of hydrazone units under basic conditions causes loss of Br^−^ from the cavity of 69.

To date the topological chirality of knots has not been exploited for enantioselective host–guest chemistry. With increasing access to enantiopure knots, as well as the ability to vary backbone elements, tightness, preorganising metals and other substituents, more selective sensors that exploit entanglement in their structure seem likely to be developed in the future.

### 4.2. Mechanical stoppering

**Fig. 33:**
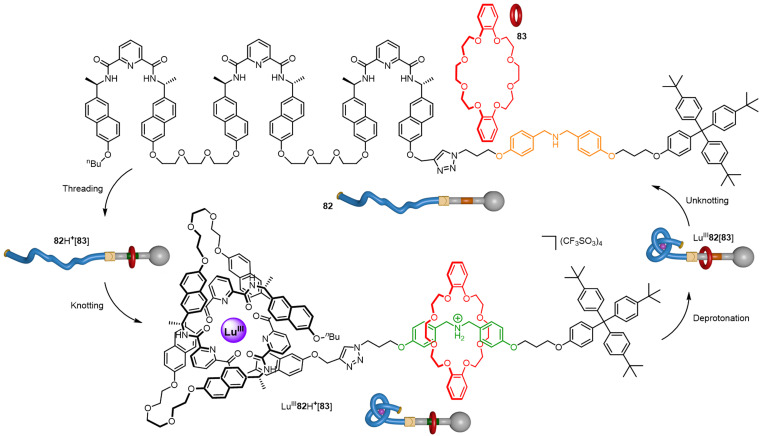
Four-step tying-untying manipulation of a molecular stopper knot which secures a pseudo-rotaxane architecture.^136^

In the macroscopic world, stopper knots are used to secure a rope through a narrow passage by preventing unreeving. At the macroscale this works due to friction which is unavailable as a force at the molecular level. However, a molecular knot can perform a similar role by acting as a steric barrier to (de)threading through a macrocyclic cavity (Fig. 33).^136^ Axle 82 features a secondary ammonium group stoppered with a trityl residue on one end of the strand and a switchable overhand knot on the other. In the absence of Lu^III^, crown ether macrocycle 83 can dynamically traverse the thread to bind to the ammonium unit to give 82H^+^[83]. Addition of Lu^III^ promotes folding to give overhand knot complex Lu^III^82H^+^[83], which acts as a steric barrier that secures the pseudo-rotaxane architecture, even when the ammonium unit is deprotonated to give Lu^III^82[83]. Subsequent disentangling of the stopper knot by removal of Lu^III^ with F^−^ causes macrocycle 83 to be spontaneously released back into solution.

### 4.3. Liquid crystal dopants

**Fig. 34:**
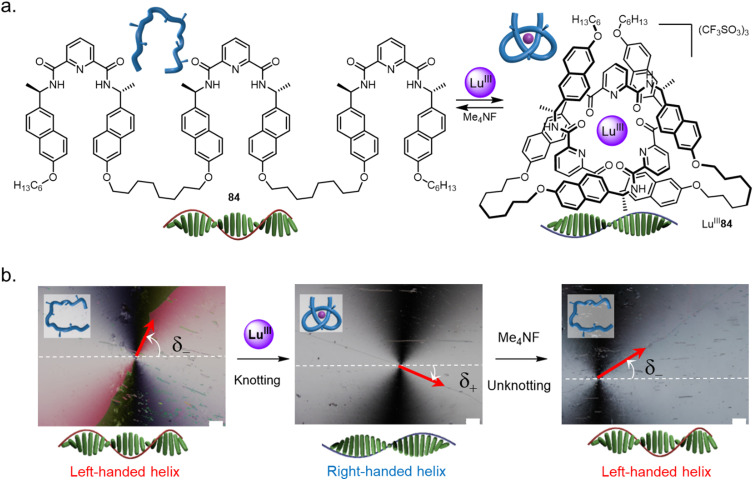
Tying a molecular knot inverts chirality expression in liquid crystals. (a) Left-handed helical liquid crystal organization results with homochiral strand 84 as a dopant, while right-handed organization occurs with the corresponding knot Lu^III^84. (b) The knot can be tied and untied *in situ* to reversibly invert the chiral expression within the liquid crystal, as evidenced by polarized optical microscopy photographs of θ-cell disclination lines.^137^ Reworked from ref. 137 with permission from the authors, copyright 2020.

Folding and entwining a molecular strand (nanometre scale) into an overhand knot can influence the structure seven orders of magnitude (centimetre scale) longer than itself.^137^ The handedness of liquid crystalline matrices was controlled by tying a knot in a homochiral molecular strand dopant 84 (Fig. 34a). When doped with strand 84 the chiral nematic liquid crystal adopts a left-handed helical twist (Fig. 34b). However, folding the strand by adding Lu^III^ causes a right-handed helical twist to be induced in the chiral nematic liquid crystal. Folding and entwining the strand overrides the expression of the point-chiral centres. The switching of the liquid crystal pitch could be performed *in situ*. Unfolding of Lu^III^84, triggered by F^−^, reset the system to once again adopt a left-handed twist.

### 4.4. Catalysis

**Fig. 35:**
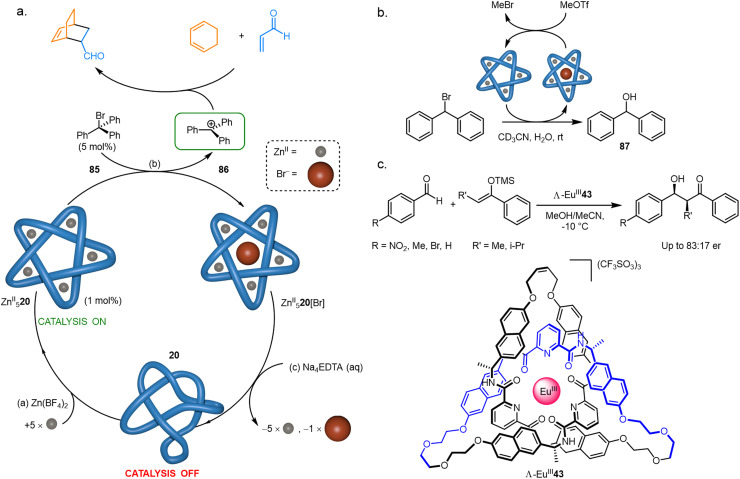
Molecular knots and catalysis (a) initiation and regulation of Lewis acid catalysis by Fe^II^_5_20. (b) Catalysis of a hydrolysis reaction by Fe^II^_5_20.^55^ (c) Enantioselective catalysis of the Mukaiyama aldol addition with Λ-Eu^III^43.^80^

It has been demonstrated that knotting a molecular strand can cause it to adopt a catalytically active conformation. The anion binding ability (Section 4.1) of pentafoil knot Zn^II^_5_20 was exploited to initiate and regulate catalysis (Fig. 35). Treatment of Zn^II^_5_20 with trityl bromide 85 generates trityl cation 86, which then catalyses Diels–Alder and Michael reactions by Lewis acid activation (Fig. 35a).^55^ The trityl cation is only generated when the knotted strand is metallated.

Pentafoil knot Zn^II^_5_20 can also accelerate chemical reactions itself: Zn^II^_5_20 abstracts Br^−^ from Ph_2_CHBr to give the corresponding carbocation, which then undergoes rapid hydrolysis to form 87 (Fig. 35b). Turnover of the knot is achieved by adding MeOTf, which reacts with knot-bound Br^−^ to give volatile MeBr, regenerating the empty knot cavity. A similar anion-binding strategy was used by Trabolsi to activate bromo-derivatives of Morita–Baylis–Hillman adducts towards hydrolysis.^138^ The use of different metals in the knot frameworks gave different rates of hydrolysis, with the Cu^II^ derivative outperforming the Zn^II^ and Cd^II^ complexes.

As many knots are chiral, it follows that they may be active in asymmetric catalysis. Λ-Eu^III^43, a trefoil knot resulting from closure of a homochiral overhand knot (Fig. 35c), was found to catalyse an asymmetric Mukaiyama aldol addition, providing high conversions and modest enantiomeric ratios of up to 83 : 17.^80^ Lanthanide luminescence decay studies of Λ-Eu^III^43 indicated that the metal cation is accessible to solvent molecules despite being buried within the knotted structure, suggesting that the catalysis likely proceeds *via* coordination of the aldehyde to the lanthanide ion bound deep within the asymmetric knot environment.

### 4.5. Ion channel formation

It has recently been shown that the anion binding ability and rigid structures of metallated pentafoil knot Fe^II^_5_20 and Star of David catenane Fe^II^_6_21 enable them to act as highly active and selective ion channels.^139^ Their complex hierarchical structures, pore-like architecture and nanoscale dimensions are reminiscent of transmembrane proteins such as hemolysin A. Phospholipid vesicles in buffered H_2_O solution (pH 7.4) were used with 8-hydroxypyrene-1,3,6-trisulfonate (HPTS) assays to determine ion channel formation and ion selectivity. Pentafoil knot Fe^II^_5_20 showed only weak channel forming activity with Br^−^ anions, presumably due to the small cavity size (Fig. 29). However, metallated Star of David Fe^II^_6_21 showed excellent ionophoric activity, with activity on the same order of magnitude as state-of-the-art synthetic ion channels. Activity followed the Hofmeister series, suggesting that desolvation was the rate-limiting factor for ion transport. The demetallated Star of David catenane displayed no ionophoric activity, consistent with its lack of anion binding in solution. Channel formation was evidenced by the observation of quantised transport events in single-channel planar bilayer conductance experiments.

### 4.6. Nanotherapeutics

**Fig. 36:**
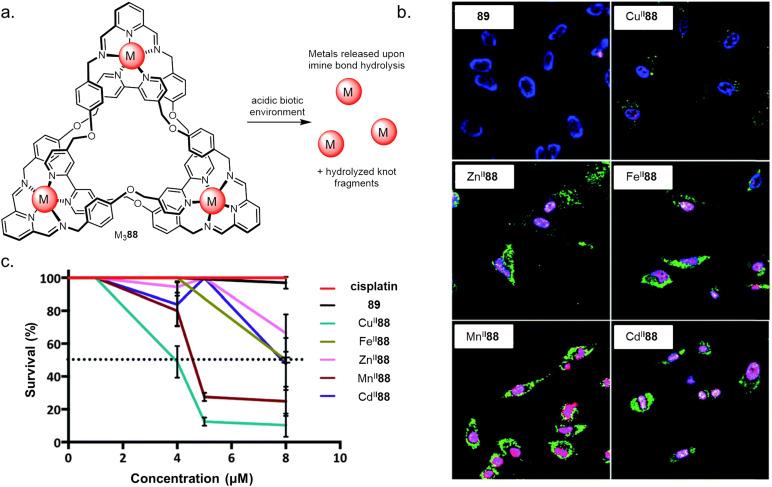
(a) The use of molecular knots as delivery vectors of metals to trigger apoptosis in live cells. (b) Confocal images of HeLa cells incubated with Cu^II^88, Zn^II^88, Fe^II^88, Mn^II^88, Cd^II^88 or metal-free reduced knot 89, followed by staining with Annexin V-FITC (green), DAPI (blue) and propidium iodide (red). (c) Dose–response curves for M^II^88, cisplatin, and 89. Dotted line indicates LD50. Reworked from ref. 140*via* open access CC BY-NC 3.0 license.^140^ Reworked from ref. 139 by a CC-BY license.

Controlled metal ion release on fragmentation of an entangled strand has been used by Trabolsi to selectively target cancerous tissue (Fig. 36).^140^ The imine bonds of several knotted complexes M^II^88 (M^II^ = Cu^II^, Zn^II^, Fe^II^, Mn^II^ or Cd^II^) hydrolyse in the hypoxic acidic environment found within cancer cells, causing metal ion release and subsequent cell death. All the knots tested showed potency against six cancer lines *in vitro* and *in vivo* in zebrafish embryos.

Mechanistic studies suggested that the high activity of the knots was due to their nanoscale size, allowing them to be taken up into cells *via* active transport rather than passive diffusion. Strand entanglement does not appear to play a role other than as a delivery vector in this system. However, in the future it may be that the chirality or charged surfaces of metal–organic knots could be used to target specific protein pockets and surfaces.

## 5. Conclusions and outlook

The last decade has seen significant advances in the nascent field of molecular nanotopology, the topological counterpart of molecular nanotechnology. Much of this can be attributed to the invention and development of new and increasingly effective strategies for accessing ordered molecular entanglements. However, the simplicity of the self-assembly procedures often belies the complexities involved in the design process. For example, the one-pot assembly of 10 organic building blocks, 5 metal ions and a chloride ion to form pentafoil knot Fe^II^_5_9 (Fig. 7) requires dynamic metal coordination, reversible covalent bond formation, C–H⋯halide interactions, stereoelectronic gauche interactions and other design elements that must act together to bring about the desired architecture.

Although the first molecular knot topology (trefoil) was synthesized in 1989, it took until 2012 to realise the second (pentafoil). However, since then a further seven prime molecular and metalla-knot topologies have succumbed to chemical synthesis along with the stereoselective synthesis of granny and square knots and three other composite knot topologies. New strategies such as folding-and-entwining, grids and Vernier template synthesis have allowed rapid synthesis of complex entanglements with up to 12 crossings. Advances in the understanding of metal–ligand coordination, characterisation techniques and instrumentation underpin these accelerated advances.

With molecular knots and links becoming accessible and the anchoring of synthetic strategies now rooted in mathematical knot theory, answers to the question ‘how?’ are becoming clearer and it is time for chemists to begin to answer ‘why?’^141^ Already it is evident that the restriction in conformation imposed by entanglement can be useful in catalysis and anion binding, and molecular weaving is a new frontier for materials fabrication, but what other uses are there? Among the many open questions in molecular nanotopology, we believe that the following are some of the most interesting and challenging:

(1) A particular knot for each function. In the macroscopic world, different types of knots have different characteristics that make them more or less suited for a given task: ‘bend knots’ provide the strongest binding between two lengths of rope; ‘hitches’ are best for tying rope around an object; and ‘loop knots’ or ‘nooses’ allow degrees of movement between the components they connect. Identifying which types of molecular knot topologies are best suited for specific functions could be important for the utility of orderly entanglements at the molecular level.

(2) Unsymmetrical knots. The synthesis of most of the knots made to date has been facilitated by exploiting their symmetry (*e.g.* through multicomponent self-assembly). With the advent of folding-and-entwining strategies, higher-order unsymmetrical knots, such as the Stevedore (6_1_) twist knot, should be within reach through rational design.

(3) Exploring the size and tightness limits of knots. Tightness and number of crossings are two primary parameters that determine the properties of a knotted strand. Quantitative understanding of how these properties change strand behaviour are still lacking.

(4) Kinetically trapping dynamic knots. Imine-based knots and metalla-knots (Sections 2.3.1 and 2.10) have dynamic backbones that allow the strand to pass through itself which is not consistent with the fundamental constraints of topology. Developing covalent capture methods for the tangled ligands of such coordination complexes could give access to kinetically stable strands, turning metal-coordination into template synthesis.

(5) Orderly entangled materials. Random entanglements are present in virtually all polymer mixtures and play a major role in their materials properties. The time is ripe to systematically introduce synthetic knots and ordered strand entanglements into materials (Section 2.11) to explore the effect that regular topological restrictions have on properties.

(6) Knots under force. Little is known about what happens when molecular level knots are subject to an applied force. Does knotting weaken the strand at the apex of the entanglement as for macroscopic knots? And if so, can the induced strain be exploited, for example in bond-breaking for synthesis?

Given the substantial progress in the synthesis of molecular knots over the last few years, open questions such as these, and many others, can start to be tackled experimentally. So, like a needle pulling thread, we confidently predict that the exploration of orderly molecular entanglements has a long, long way to run.

## Conflicts of interest

There are no conflicts to declare.

## Supplementary Material
